# Pax3/7 regulates neural tube closure and patterning in a non-vertebrate chordate

**DOI:** 10.3389/fcell.2022.999511

**Published:** 2022-09-12

**Authors:** Kwantae Kim, Jameson Orvis, Alberto Stolfi

**Affiliations:** School of Biological Sciences, Georgia Institute of Technology, Atlanta, GA, United States

**Keywords:** Pax3/7, pax3, pax7, ciona, tunicate, neural tube closure, neural plate borders

## Abstract

Pax3/7 factors play numerous roles in the development of the dorsal nervous system of vertebrates. From specifying neural crest at the neural plate borders, to regulating neural tube closure and patterning of the resulting neural tube. However, it is unclear which of these roles are conserved in non-vertebrate chordates. Here we investigate the expression and function of Pax3/7 in the model tunicate *Ciona.* Pax3/7 is expressed in neural plate border cells during neurulation, and in central nervous system progenitors shortly after neural tube closure. We find that separate *cis-*regulatory elements control the expression in these two distinct lineages. Using CRISPR/Cas9-mediated mutagenesis, we knocked out *Pax3/7* in F0 embryos specifically in these two separate territories. *Pax3/7* knockout in the neural plate borders resulted in neural tube closure defects, suggesting an ancient role for Pax3/7 in this chordate-specific process. Furthermore, knocking out *Pax3/7* in the neural impaired Motor Ganglion neuron specification, confirming a conserved role for this gene in patterning the neural tube as well. Taken together, these results suggests that key functions of Pax3/7 in neural tube development are evolutionarily ancient, dating back at least to the last common ancestor of vertebrates and tunicates.

## Introduction

**FIGURE 1:**
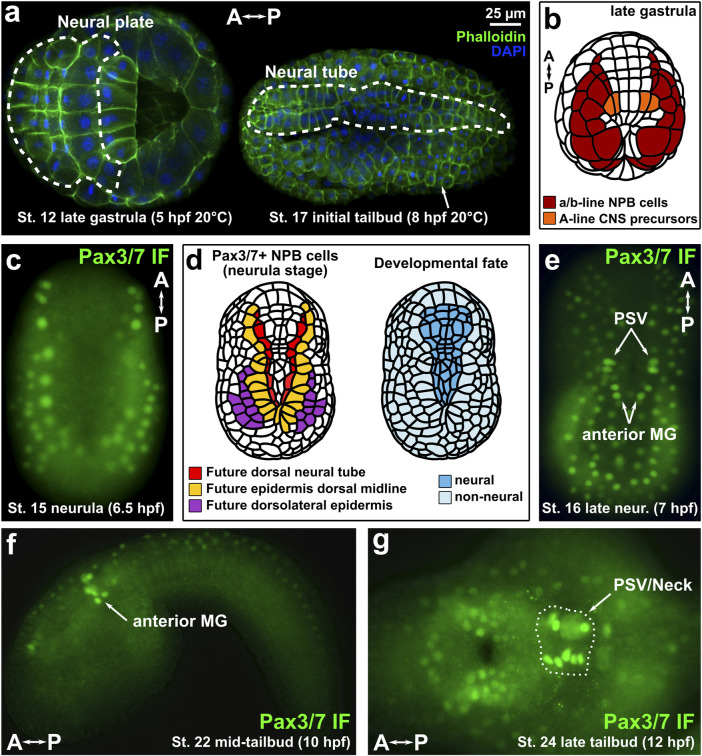
Immunofluorescence of Pax3/7 in Ciona embryos. **(A)** Ciona robusta (intestinalis type A) embryos showing neural plate and neural tube cells. **(B)** Diagram of late gastrula Ciona embryo showing Pax3/7-expressing territories examined in this study. NPB: Neural Plate Borders. CNS: Central Nervous System. **(C)** Immunofluorescence (IF) of Pax3/7 (DP312 antibody) showing expression in lateral borders of the neural plate. **(D)** Diagram of Pax3/7 + cells in the neural plate borders, indicating their ultimate contribution tothree different dorsal derivatives of the neural/non-neural boundary region of the ectoderm: dorsal neural tube, dorsal midline epidermis, and dorsolateral epidermis. **(E)** IF of Pax3/7 at the late neurula/initial tailbud stage in which specific Motor Ganglion (MG) and Posterior Sensory Vesicle (PSV) neural progenitors are labelled by anti-Pax3/7 DP312 antibody. **(F)** IF of Pax3/7 in the anterior MG of the mid-tailbud embryo. **(G)** IF of Pax3/7 at late tailbud stage, showing expression in the brain and “Neck” regions of the CNS (circled by dashed line). A–P: anterior-posterior axis.

Tunicates are marine non-vertebrate chordates and comprise the sister group to the vertebrates (Delsuc et al., 2006; Putnam et al., 2008). Work on diverse tunicate species such as *Ciona* spp. have contributed to our understanding of chordate evolution and the evolutionary origins of many vertebrate innovations (Lemaire, 2011; Satoh, 2013). In vertebrates, the lateral borders of the neural plate give rise to some of these important vertebrate innovations, such as neural crest cells and placodes. Given their importance to vertebrate development, the evolutionary origins of vertebrate neural crest and placodes have been the subject of much interest (Baker and Bronner-Fraser, 1997; Wada, 2001; Rothstein and Simoes-Costa, 2022). In cephalochordates and tunicates, a dorsal neural plate also gives rise to a hollow neural tube in a process that is similar to vertebrate neurulation (Nicol and Meinertzhagen, 1988; Albuixech-Crespo et al., 2017) (Figure 1A). The lateral borders of the neural plate (and later the dorsal part of the neural tube) in these non-vertebrate chordates also give rise to putative homologs of certain cell types that are derived from placodes and/or neural crest in vertebrates, like for instance melanin-containing pigment cells and sensory neurons (Holland et al., 1999; Yu et al., 2008; Abitua et al., 2012; Stolfi et al., 2015). A more recent model has been proposed wherein the olfactorian ancestor (last common ancestor of tunicates and vertebrates) had a placode-like embryonic territory flanking the neural plate giving rise to sensory neurons (Horie et al., 2018; Papadogiannis et al., 2022). Later elaboration of the neural plate borders would have resulted in more specialized neural crest and placodes in vertebrates.

Whatever the exact evolutionary history of neural crest and placodes, it has become clear that the lateral borders of the neural plate has been an important source of evolutionary novelties in chordate evolution. Several transcription factors show conserved, overlapping expression patterns that define these borders, among them Zic, Tfap2, Msx, and Pax3/7 (Thawani and Groves, 2020). Of these, Pax3/7 factors are arguably the most specific to the neural plate borders themselves. In vertebrates, Pax3 and Pax7 play diverse roles in the specification of neural crest cells and placodes (Maczkowiak et al., 2010; Milet and Monsoro-Burq, 2012). They are also required for neural tube closure and patterning of the dorsal neural tube, which arises from the neural plate borders in vertebrates (Epstein et al., 1991; Mansouri and Gruss, 1998). Thus, Pax3/7 factors are indispensable for neural plate border specification and later development of diverse neural crest-, placode-, and neural tube-derived cell types in vertebrates.

Given the importance of Pax3/7 factors in neural plate border specification in vertebrates, we sought to study its potentially conserved functions in *Ciona.* Understanding the role of Pax3/7 in *Ciona* might shed light on the potentially ancestral functions of this important factor in the last common chordate ancestor, predating the origins of vertebrate neural crest and placodes. In tunicates*,* a conserved *Pax3/7* ortholog is expressed in the neural plate borders of the embryo (Wada et al., 1997; Mazet et al., 2005; Stolfi et al., 2015). In the stereotyped, miniature embryos of *Ciona* and *Halocynthia roretzi,* this means that Pax3/7 expression is observed at the neurula stage as invariant anterior-posterior rows of cells fated to form the dorsal row of the neural tube and the dorsal and dorsolateral cells of the epidermis, prior to neural tube closure. Additionally, Pax3/7 is also expressed in progenitor cells located in the lateral rows of the neural tube after its closure. More specifically, this includes the anterior part of the Motor Ganglion (MG), which was proposed to be homologous to dorsal parts of the rhombospinal regions of the vertebrate nervous system (Stolfi et al., 2011). Although in vertebrates the dorsal neural tube is derived from the neural plate borders, this is not the case in *Ciona,* in which Pax3/7+ MG precursors are not derived from Pax3/7+ neural plate border cells but rather from an entirely different lineage altogether (Nicol and Meinertzhagen, 1988; Cole and Meinertzhagen, 2004; Imai et al., 2009) (Figure 1B). Thus, while Pax3/7+ neural plate border cells are derived from the animal pole of the *Ciona* embryo (a/b-lineages), Pax3/7+ MG cells are derived from the vegetal pole (A-lineage).

Here we use tissue-specific CRISPR/Cas9 to investigate the function of *Pax3/7* in these different cell lineages that are evolutionarily linked to the vertebrate neural plate border and its derivatives. We show that knocking out *Pax3/7* in the neural plate borders impairs neural tube closure, as well as specific gene expression. *Pax3/7* knockout in the MG also confirmed its crucial role in patterning this compartment and specifying commissural neurons similar to its role in the vertebrate dorsal spinal cord. These results show that, although tunicates lack conventional neural crest and cranial placodes, conserved Pax3/7-dependent programs for neural plate border specification, neural tube closure, and motor circuit patterning likely predate the origin of vertebrates.

## Methods

### Immunofluorescence of Pax3/7

Monoclonal antibodies DP311 and DP312, raised against *Drosophila* Prd (Davis et al., 2005) were kindly provided by Nipam Patel. Embryos were fixed in MEM-FA (3.7% formaldehyde, 0.1 M MOPS pH7.4, 0.5 M NaCl, 1 mM EGTA, 2 mM MgSO4, 0.05% Triton X-100), washed/quenched in 1X PBS, 0.35% Triton X-100, 50 mM NH4Cl, and washed in 1X PBS, 0.05% Triton X-100. Embryos were then blocked for 30 min at room temperature in “Blocker” Buffer (1X PBS, 0.05% Triton X-100, 1% Thermo Scientific “Blocker” BSA). Embryos were incubated in antibodies 1:100 in “Blocker” Buffer overnight at 4°C. Embryos were washed three times in 1X PBS, 0.05% Triton X-100 (rocking 15 min at room temperature, each time). Samples were incubated in AlexaFluor-488 anti-mouse IgG secondary antibody 1:500 in Blocker buffer for 1 h at room temperature. Washes were performed as in the previous step before mounting and imaging.

### Embryo electroporations

Gravid adult *Ciona robusta (intestinalis Type A)* were collected and shipped by M-REP from San Diego, CA. Embryo dechorionation and electroporation was performed as previously established (Christiaen et al., 2009a, Christiaen et al., 2009b). Fluorescent reporter plasmids were electroporated at concentrations of 10–35 µg/700 µl electroporation volume for histone fusions, and 70–100 µg/700 µl for other reporters. Leech H2B::mCherry and Unc-76::GFP/YFP reporter plasmid backbones have been previously published (Gline et al., 2009; Imai et al., 2009). *Tyrp. a>2XGFP* was a kind gift from Filomena Ristoratore (Racioppi et al., 2014). Embryos were fixed and washed as for immunofluorescence above, without blocking or incubating. Embryos were mounted in Mounting Solution (1X PBS, 2% DABCO, 50% Glycerol) and imaged on compound epifluorescence or scanning-point confocal microscopes. All relevant sequences can be found in Supplementary Material S1.

### CRISPR/Cas9

*Pax3/7* sgRNA expression cassettes on the “F + E” optimized scaffold (Chen et al., 2013; Stolfi et al., 2014) were designed by CRISPOR (Haeussler et al., 2016) and cloned together with the U6 promoter (Nishiyama and Fujiwara, 2008) by One-Step Overlap PCR (OSO-PCR) as previously described (Gandhi et al., 2017; Gandhi et al., 2018). OSO-PCR cassettes were individually tested for mutagenesis efficacy by the “peakshift” method (Gandhi et al., 2018) before cloning into plasmids, by electroporating 25 µl of unpurified PCR product and 25 µg of Eef1α>Cas9 (Sasakura et al., 2010; Stolfi et al., 2014) per 700 µl volume (Supplementary Table S1). Amplicons representing alleles from pools of embryos were PCR amplified and Sanger-sequenced as detailed in Gandhi et al., 2018. All sgRNA, Cas9 vector, and primer sequences and electroporation recipes are in Supplementary Material S1.

## Results

### Immunofluorescence of Pax3/7

To visualize Pax3/7 expression in the neural plate borders of *C. robusta* (also known as *intestinalis Type A*), we performed immunofluorescence (IF) staining of neurula- and tailbud-stage embryos using monoclonal antibodies raised against the *Drosophila* Pax3/7 ortholog (Paired), which show broad specificity for pan-metazoan Pax3/7 proteins (Davis et al., 2005). Both antibody clones DP311 and DP312 showed staining of nuclearly-localized Pax3/7 expressed in anterior-posterior rows of cells at the mid-neurula stage, which will contribute to dorsal neural tube and dorsal midline/dorsolateral epidermis later on (Figures 1C,D, Supplementary Figure S1) (St. 15; 6.5 h post-fertilization or hpf at 20°C), consistent with its expression by mRNA *in situ* hybridization as previously reported in *Ciona* (Stolfi et al., 2015) and in the nearly identical embryos of another tunicate species, *Halocynthia roretzi* (Ohtsuka et al., 2014). Although DP311 showed qualitatively less background, DP312 staining was brighter overall, so we proceeded with the latter clone. At late neurula stage (St. 16; 7 hpf at 20°C), staining started to appear in the progenitors of the posterior sensory vesicle (PSV) region of the brain, and anterior MG (Figure 1E). At mid-tailbud stage (St. 22; 10 hpf at 20°C), Pax3/7 IF signal was observed in the lateral rows of the anterior MG (Figure 1F), while slightly later at St. 24 (12 hpf, 20°C) signal was stronger in the PSV and “neck”, which are neural progenitors situated between the neurons of the brain and the MG (Figure 1G). These patterns are also consistent with those detected by *Pax3/7 in situ* hybridization previously (Imai et al., 2009; Stolfi et al., 2011).

### *Cis-*regulatory modules controlling *Pax3/7* expression

**FIGURE 2:**
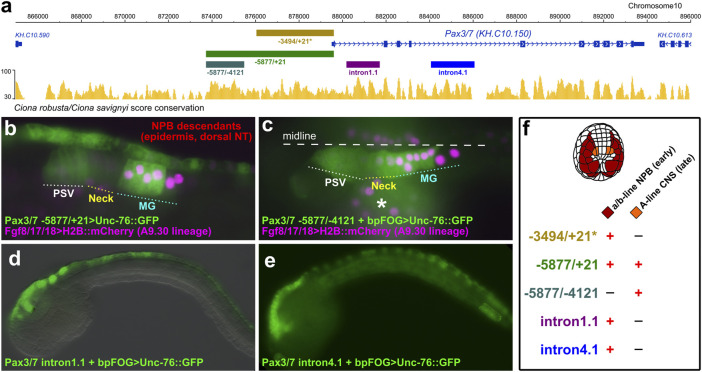
Pax3/7 reporter plasmids. **(A)** Diagram from ANISEED genome browser (Dardaillon et al., 2020), showing location of *cis*-regulatory sequences and conservation between *Ciona robusta/savignyi.* Asterisk denotes -3494 upstream fragment previously published (Horie et al., 2018). **(B)** Late tailbud (St. 23) embryo electroporated with *Pax3/7–5877/+21>Unc-76::GFP* and *Fgf8/17/18>H2B::mCherry* (which labels the A9.30 lineage that gives rise to most of the MG)*,* showing expression in neural plate border (NPB) descendants in the dorsal midline of the epidermis and neural tube, and in compartments of the central nervous system (CNS) including Posterior Sensory Vesicle (PSV) and Motor Ganglion (MG). **(C)** St. 23 embryo electroporated with *Pax3/7–5877/-4121 + bpFOG>Unc-76::GFP* and *Fgf8/17/18>H2B::mCherry,* showing expression only in the CNS, not in the NPB descendants. Asterisk indicates mesenchyme, which shows leaky expression of reporters. **(D)** Embryo electroporated with *Pax3/7 intron1.1 + bpFOG>Unc-76::GFP.* This partial intronic fragment drives reporter gene expression in the descendants of the NPBs. **(E)** Embryo electroporated with *Pax3/7 intron4.1 + bpFOG>Unc-76::GFP.* This partial intronic fragment also drives expression in part of the NPB descendants. **(F)** Summary of expression observed with the different *Pax3/7* reporter constructs, indicating expression in a/b-line and A-line cells. bpFOG = basal promoter of *FOG,* which is used as a minimal promoter to test isolated *cis*-regulatory elements in *Ciona.* Unc-76::GFP = GFP tagged with an Unc-76 fragment used to exclude GFP from the nucleus and to completely fill axons, routinely used in *Ciona*.

Because the early (neurula) and late (tailbud) domains of Pax3/7 expression are not connected by descent, arising from distinct blastomeres of the 8-cell stage embryo, we hypothesized that separate *cis*-regulatory modules might control their activation in the different lineages. Previously, a ∼3.5 kb sequence immediately upstream of the *Pax3/7* transcription start site had been shown to drive reporter gene expression in the neural plate borders (Horie et al., 2018) (Figure 2A). We tested a longer sequence stretching further upstream, comprising -5877 bp upstream of the Pax3/7 start codon, alongside the *Fgf8/17/18* reporter which labels the A9.30 lineage that gives rise to most of the MG (Imai et al., 2009) The extended fragment was sufficient to drive GFP reporter gene expression in both the neural plate border derivatives and the lateral rows of the neural tube in the brain, neck, and anterior MG (Figure 2B). Expression in the Neck was often not as bright as in the brain or MG, reflecting perhaps the apparent downregulation in this compartment as observed by *in situ* previously (Imai et al., 2009). When we isolated just the -5877 to -4121 fragment and placed this in front of the basal promoter of the *Friend of GATA* gene (bpFOG), which is routinely used in *Ciona* as a minimal promoter, (Rothbächer et al., 2007), this was sufficient to drive GFP expression in the brain/neck/MG but not in the neural plate borders (Figure 2C). Expression was seen in both A11.120 and A11.119 left/right pairs of neural progenitor cells in the MG. Although expression of *Pax3/7* in A11.119 appears to be downregulated when observed by *in situ* hybridization (Stolfi et al., 2011), it has been reported as initiating in the mother cell (A10.60). Thus, this likely represents GFP protein accumulation, and is consistent with the IF staining observed at 10 hpf (Figure 1E).

We also identified separate *cis*-regulatory elements in introns 1 and 4 that were also sufficient to drive expression in neural plate border derivatives (Figures 2D,E), hinting at complex regulatory control of *Pax3/7* expression through partially overlapping “shadow enhancers” (Hong et al., 2008). Taken together, our results suggest that the different domains of *Pax3/7* expression in the neurectoderm (neural plate borders and lateral rows of the brain/neck/MG) are largely regulated by distinct *cis*-regulatory elements (Figure 2F).

### CRISPR/Cas9-mediated knockout of *Pax3/7*

**FIGURE 3:**
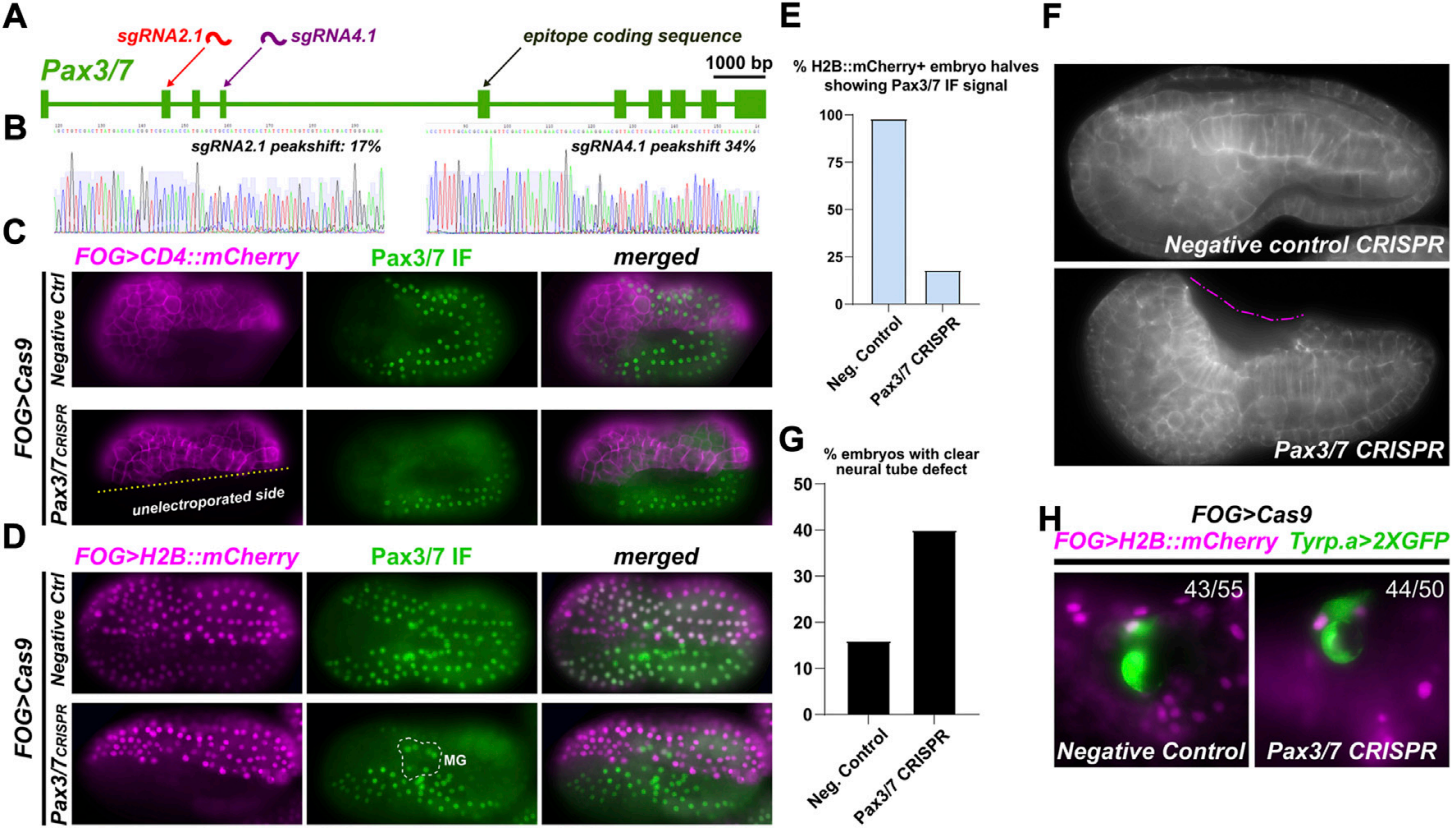
*Pax3/7* CRISPR in the neural plate borders. **(A)** Diagram of *Pax3/7* locus and location of sgRNA targets, relative to epitope sequence recognized by DP312 antbody. **(B)** Peakshift sequencing traces indicating presence of indels resulting from CRISPR/Cas9-mediated double-stranded DNA breaks. **(C)** Tissue-specific CRISPR/Cas9-mediated knockout of *Pax3/7,* performed in a/b-line cells using the *FOG>Cas9* plasmid and assayed by DP312 immunofluorescence at stage 16. *FOG>CD4::mCherry* counterstains cell membranes, demonstrating the specificity and efficacy of *Pax3/7* knockout. **(D)** *Pax3/7* CRISPR and negative control embryos generated as above (same stage also), but using *FOG>H2B::mCherry* to score proportion of CRISPR knockouts. Motor Ganglion (MG) cells are not derived from a/b lineages, and therefore Cas9 is never expressed in their lineage. Thus, Pax3/7 signal is not lost from the MG, only NPBs. **(E)** Embryos scored for loss of Pax3/7 immunofluorescent signal (from panel d), showing substantial loss of staining in *Pax3/7* CRISPR H2B::mCherry+ NPB cells (n = 40 embryo halves) compared to negative control (n = 43 halves). **(F)** *Pax3/7* CRISPR and negative control (U6>DenhT2) embryos stained with fluorescent phalloidin conjugate to reveal cell outlines after neural tube closure, at stage 20. Dashed pink line denotes a large cavity as a result of failed neural tube closure. **(G)** Embryos above were scored for incomplete neural tube closure defect (n = 100 embryos each condition). Embryos with inconclusive neural tube closure phenotype were not scored as defective. **(H)** *Pax3/7* CRISPR does not affect the specification of neural plate border-derived pigment cells. Ratios indicate number of FOG>H2B::mCherry+ larvae (19 hpf, 20°C) showing *Tyrp.a>2XGFP* reporter expression (43 of 55 larvae in negative control, 44 of 50 larvae in *Pax3/7* CRISPR).

To study the functions of *Pax3/7* in neural development in the tunicate embryo*,* we sought to use tissue-specific CRISPR/Cas9 as previously adapted to *Ciona* (Sasaki et al., 2014; Stolfi et al., 2014; Gandhi et al., 2017). We tested two candidate single-chain guide RNA (sgRNA) constructs (Pax3/7.2.1 and Pax3/7.4.1), targeting exons 2 and 4 respectively (Figure 3A). We validated their mutagenesis efficacies following the “peakshift” method of Sanger-sequencing PCR amplicons of targeted sequences (Gandhi et al., 2018). Efficacies for Pax3/7.2.1 and Pax3/7.4.1 were 17% and 34% mutagenesis, respectively (Figure 3B) (Supplementary Table S1).

We next tested the ability of these sgRNAs, when combined, in eliminating Pax3/7 expression from the neural plate borders. To do this, we co-electroporated the sgRNAs with *FOG>Cas9,* which drives Cas9 expression in all animal pole-derived cells including the neural plate borders. A *FOG>CD4::mCherry* reporter plasmid was also co-electroporated to reveal transfected cells’ outlines, and DP312 antibody IF was used to assay Pax3/7 expression. Pax3/7 sgRNAs were compared to a negative control sgRNA (“Control”) that does not target any sequence in the *Ciona* genome (Stolfi et al., 2014). While in the negative control neurula embryos (St. 16) the Pax3/7 antibody clearly labeled the nuclei of both transfected and non-transfected cells (Figure 3C), we detected substantial loss of Pax3/7 IF signal in cells transfected with *Pax3/7* CRISPR constructs. Non-transfected cells in the same embryos served as a clean internal control for Pax3/7 IF, showing that the loss of Pax3/7 was visibly confined to only transfected cells. In some embryos, no Pax3/7+ cells were seen at all in the transfected half, suggesting biallelic knockout of *Pax3/7*.

To score this, we performed Pax3/7 IF on *Pax3/7* CRISPR and negative control embryos co-electroporated *FOG>H2B::mCherry* to visualize the nuclei of transfected cells at stage 16 (Figure 3D; Supplementary Figure S2). Cells were scored for H2B::mCherry expression and Pax3/7 IF signal, independently on either left or right borders of the neural plate to account for mosaicism (Figure 3E). Embryos with no H2B::mCherry expression in the neural plate borders (untransfected) were not included, as were embryos oriented in a way that obscured the view of the neural plate borders. In 52 such *Pax3/7* CRISPR embryo halves, 40 were H2B::mCherry+, but only 7 of those were also positive for Pax3/7 while 33 were negative, indicating loss of Pax3/7 in 82% of transfected neural plate borders. In negative control embryos, 42 of 43 H2B::mCherry + halves were positive for Pax3/7. Pax3/7 was observed in 100% of untransfected (H2B::mCherry-negative) neural plate border cells in both *Pax3/7* CRISPR (12/12) and negative control embryos (10/10). In sum, these results confirmed the specificity and high efficacy of *Pax3/7* CRISPR knockout in this system.

### Knockout of *Pax3/7* in the neural plate borders impairs neural tube closure

In *Ciona* and vertebrates, neural tube closure is driven in part by epithelial “zippering” involving the formation of cellular “rosettes” in which cells undergo sequential apical contraction and cell junction exchange (Hashimoto et al., 2015; Hashimoto and Munro, 2019; Mole et al., 2020). In some of our CRISPants, we noticed a lack of such rosettes and epithelial zippering, implying that perhaps loss of *Pax3/7* might impair this process (Figure 3C; Supplementary Figure S3). In vertebrates, Pax3/7 factors are essential for neural tube closure (Epstein et al., 1991). Thus, we sought to determine if CRISPR knockout of *Pax3/7* might induce similar neural tube defects in *Ciona.*

We carried out tissue-specific knockout of *Pax3/7* as described above, targeting Cas9 and therefore CRISPR activity to the animal pole (neural plate borders, not brain/neck/MG). We then imaged early tailbud embryos (St. 20) looking for any neural tube closure defects, not differentiating between mild or severe defects (Figure 3F). When we scored *Pax3/7* CRISPR embryos (Figure 3G), we observed defects in 40% of embryos (n = 100). 36% had seemingly normal neural tube closure, while 22% of embryos were “unclear” due to orientation of embryo on the slide. In contrast, in embryos electroporated with the same components except a negative control sgRNA (“DenhT2”) in the place of *Pax3/7-*specific sgRNAs, only 16% embryos showed a neural tube defect of any severity, likely due to non-specific effects of dechorionation/electroporation. 59% of negative control embryos were normal, while 25% were unclear. These data suggest that, as in vertebrates, Pax3/7 is required in the neural plate borders for normal neural tube closure.

To test whether Pax3/7 regulates conserved gene expression and/or specifies conserved cell types derived from the neural plate borders in *Ciona*, we focused on the melanin-containing pigment cells that give rise to the ocellus and otolith pigment cells. These two cells arise from the neural plate borders and might be evolutionarily linked to neural crest-derived melanocytes in vertebrates (Abitua et al., 2012; Olivo et al., 2021). However, *Pax3/7* CRISPR did not significantly affect their specification, as assayed by expression of the *Tyrp.a>2XGFP* reporter (Racioppi et al., 2014). Therefore, we cannot conclude whether Pax3/7 is required or not for the specification and differentiation of neural plate border-derived cell types in *Ciona.*

### MG-specific knockout of *Pax3/7* blocks ddN specification

**FIGURE 4:**
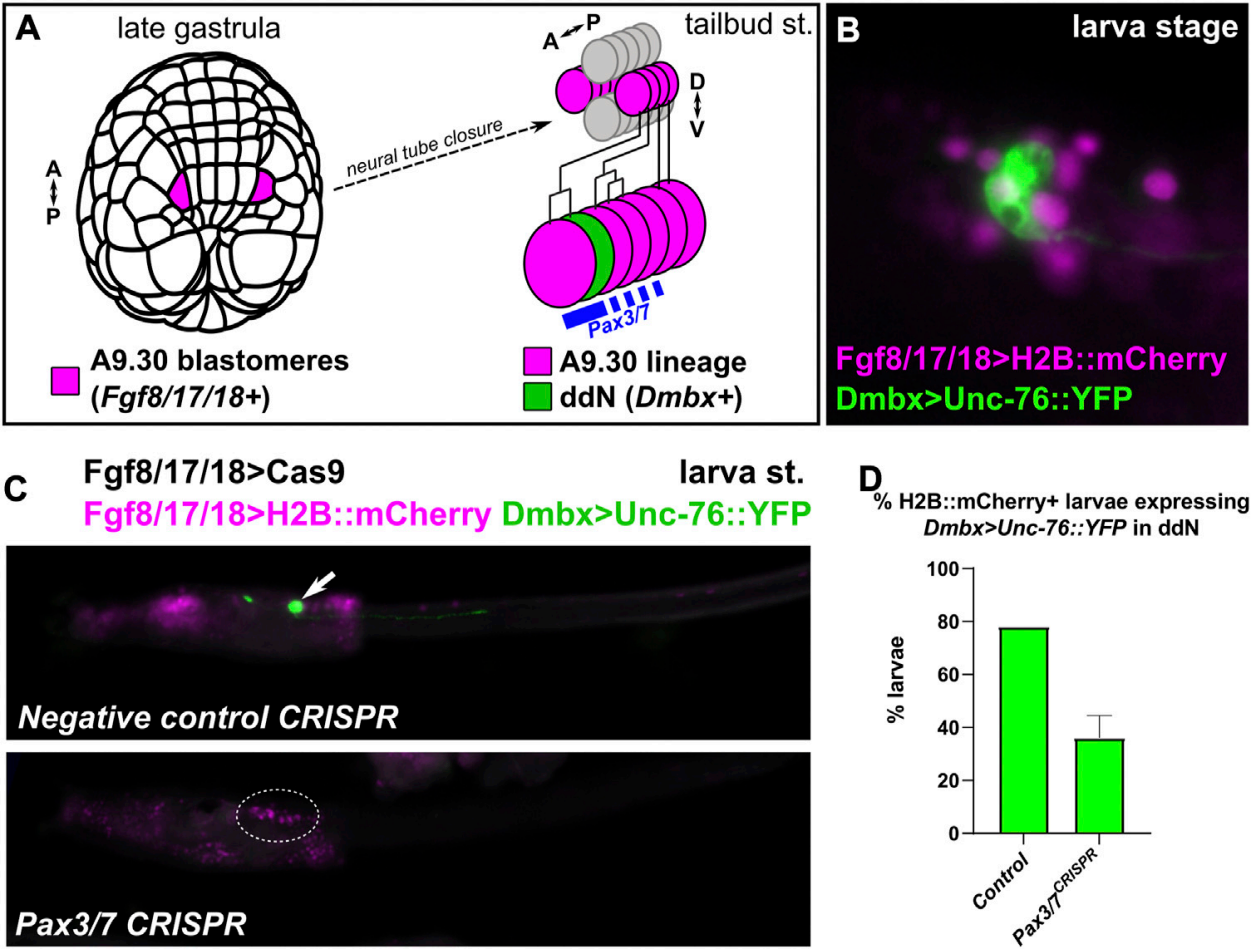
*Pax3/7* CRISPR in the Motor Ganglion. **(A)** Diagram indicating the descent of the A9.30 pair of blastomeres which give rise to the Pax3/7+ cells of the anterior Motor Ganglion (MG), including the *Dmbx*-expressing descending decussating neuron (ddN). A–P: anterior-posterior, D–V: dorsal-ventral. **(B)** Larva electroporated with *Fgf8/17/18>H2B::mCherry* and *Dmbx > Unc-76::YFP,* showing the Dmbx+ ddN cell. **(C)** A9.30 lineage-specific CRISPR/Cas9-mediated knockout of *Pax3/7* using *Fgf8/17/18>Cas9* to limit CRISPR activity to the A9.30 lineage, avoiding other territories (e.g. neural plate borders). *Dmbx* reporter plasmid labels ddNs (arrow) in negative control larvae, but this labeling is eliminated (dotted outline) in a large proportion of *Pax3/7* CRISPR larvae. **(D)** Larvae at left were scored for presence/absence of *Dmbx* reporter expression in the ddNs. Experiment was performed in duplicate, n = 50 for each condition per replicate. Error bars indicate range.

Although its role in specifying cells derived from the animal pole-derived lineages of the neural plate borders of *Ciona* remains unclear*,* Pax3/7 was previously shown to specify anterior MG fates derived from more medial cells of the neural plate. Pax3/7 is required for the specification of a single pair of descending decussating neurons (ddNs, A12.239 cell pair), which was shown through a combination of morpholino knockdown (Imai et al., 2009), overexpression of full-length Pax3/7 and dominant-repressor (Pax3/7::WRPW) as well as *cis*-regulatory analyses (Stolfi et al., 2011). However, a genetic knockout of *Pax3/7* was never attempted in the *Ciona* MG. We used the *Fgf8/17/18* promoter (Imai et al., 2009) to drive expression of Cas9 in the A9.30 lineage, which gives rise to most of the core MG including the ddNs (Figure 4A). *Fgf8/17/18>Cas9* was co-electroporated with *Pax3/7-*targeting sgRNAs and a *Dmbx* reporter plasmid (Figure 4B) (Stolfi and Levine, 2011), and larvae were assayed for reporter gene expression in ddNs. As predicted, an average of 36% of *Pax3/7* CRISPR larvae showed *Dmbx* reporter expression across two replicates, compared to an average of 78% of negative control CRISPR larvae (Figures 4C,D). These results further confirm the requirement of Pax3/7 in the regulation of *Dmbx* expression and ddN specification in the *Ciona* MG.

## Discussion

Here we show that the main ortholog of vertebrate *Pax3* and *Pax7* in tunicates, *Pax3/7,* plays conserved roles in neural development. In vertebrates, *Pax3* and *Pax7* have overlapping expression patterns and functions, which evolved following duplication of a single ancestral *Pax3/7* gene (Wada et al., 1997). While *Ciona* also has a divergent, tunicate-specific *Pax3/7* paralog which has been named *Pax3/7-related* (ANISEED gene ID: Cirobu.g00006874), that lacks the paired box domain and is not significantly expressed during embryogenesis (Imai et al., 2004). Expression of *Pax3/7* in the neural plate borders of tunicates is interesting as it implies specific ancestral functions that predate the emergence of neural crest in vertebrates, which arise from this territory and depend on Pax3/7 for their specification (Monsoro-Burq, 2015).

We have shown that *Pax3/7* is expressed in the lateral borders of the neural plate in *Ciona* and is required there for proper neural tube closure of *Ciona*, as shown by our CRISPR/Cas9-mediated knockout. Nodal signaling has been previously implicated in neural tube closure in *Ciona* (Mita and Fujiwara, 2007). One of the direct transcriptional targets of the Nodal pathway in the neural plate borders is *Pax3/7* (Mita et al., 2010). Therefore, the effects of Nodal perturbation on neural tube closure observed might be effected in part by Pax3/7 function. Pax3 mouse mutants show frequent neural tube defects (Epstein et al., 1991; Greene et al., 2009; Sudiwala et al., 2019), and mutations in the human *PAX3* gene have been found in a small number of individuals with neural tube defects (Hart and Miriyala, 2017). In mouse, supplementation of folic acid suppresses the incidence of neural tube defects in Pax3 mutants, suggesting that understanding Pax3 function is key to understanding how folic acid works to prevent neural tube defects in human development (Burren et al., 2008). However, little is known about how the transcriptional targets of Pax3 contribute to neural tube closure. *Ciona* embryos have been shown to be a powerful model for studying the cellular dynamics of neural tube closure, especially the process for epithelial zippering (Hashimoto et al., 2015; Hashimoto and Munro, 2019). Given the defects observed in this process upon CRISPR/Cas9-mediated knockout of *Pax3/7,* we propose that *Ciona* might also be a good model in which to study potentially conserved effectors of neural tube closure.

We were not able to answer whether or not Pax3/7 is required for the specification of conserved cell types arising from the neural plate borders in *Ciona,* nor what its transcriptional targets might be in this territory. In *Halocynthia,* injected *Pax3/7* mRNA was previously shown to be sufficient to activate ectopic pigment cell gene expression (Wada et al., 1997). This discrepancy might be due to species differences, or might point to Pax3/7 being sufficient, but not necessary, for pigment cell specification in tunicates. Future studies will be required to identify the targets of Pax3/7 in the tunicate neural plate borders and compare them to Pax3/7 targets in vertebrates, especially genes required for the specification of cell types derived from neural crest. One possibility is that role of Pax3/7 in the development of neural crest-derived cell types (e.g. melanocytes) is a vertebrate innovation, even if their developmental origins from the neural plate borders might predate the appearance of neural crest and vertebrates themselves.

Finally, we show that *Ciona* Pax3/7 is required later on for the specification of neurons in the anterior MG, which is proposed to be homologous to vertebrate dorsal spinal cord and hindbrain (Stolfi et al., 2011; Ryan et al., 2017). In vertebrates, there is lineage continuity between the neural plate borders and the dorsal neurons of the spinal cord and hindbrain. It was shown that separate *cis*-regulatory elements control the onset and maintenance of *Pax3* expression in the vertebrate neural plate borders/dorsal neural tube (Moore et al., 2013), and it was proposed that these separate elements control onset and maintenance in the same cell lineage (Moore et al., 2013). In contrast, the *Pax3/7-*expressing cells of the neural plate border (a/b-lineage) do not give rise to later *Pax3/7-*expressing MG neurons (A-lineage) in *Ciona* (Imai et al., 2009). We propose that the physical and ontological separation between rhombospinal neural progenitors and neural plate borders is a tunicate-specific innovation. *Pax3/7* expression would have been split into these two separate lineages through the elaboration of two separate *cis-*regulatory elements. It is possible that the last common ancestor had separate elements for onset and maintenance of *Pax3/7* expression, and that in tunicates the “onset” element became dedicated exclusively to earlier neural plate border expression, while the “maintentance” element became dedicated to later neural tube expression. Further analysis of these different *cis-*regulatory elements will be needed to refine this evolutionary model.

## Data Availability

The original contributions presented in the study are included in the article/Supplementary Materials, further inquiries can be directed to the corresponding author.
