# Supplementary material for: Pax3/7 regulates neural tube closure and patterning in a non-vertebrate chordate
Source: Front Cell Dev Biol. 2022 Sep 12;10:999511. doi: 10.3389/fcell.2022.999511 (PMC9511217; doi:10.3389/fcell.2022.999511)
Supplement: Supplementary file 1 [file Table1.DOCX]

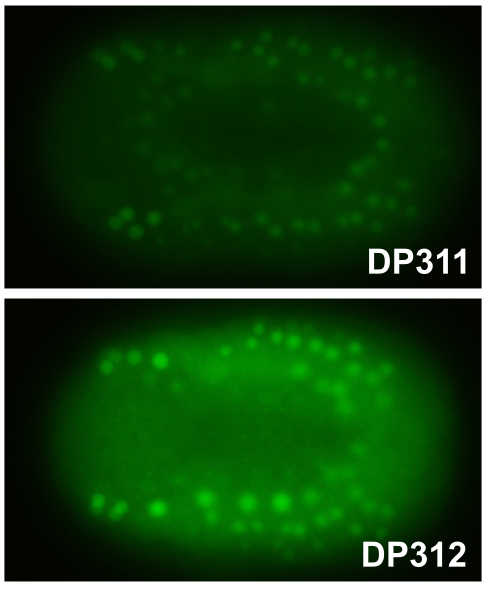


**Supplementary Figure 1. Pax3/7 antibody testing.**

Immunofluorescent staining of Stage 15 neurula embryos using two anti-Pax3/7 antibodies generously shared by Nipam Patel (Davis et al. 2005). AlexaFluor488-Mouse IgG (Thermo Scientific) was used for secondary antibody.
