# Supplementary material for: Pax3/7 regulates neural tube closure and patterning in a non-vertebrate chordate
Source: Front Cell Dev Biol. 2022 Sep 12;10:999511. doi: 10.3389/fcell.2022.999511 (PMC9511217; doi:10.3389/fcell.2022.999511)
Supplement: Supplementary file 2 [file Table2.DOCX]

**
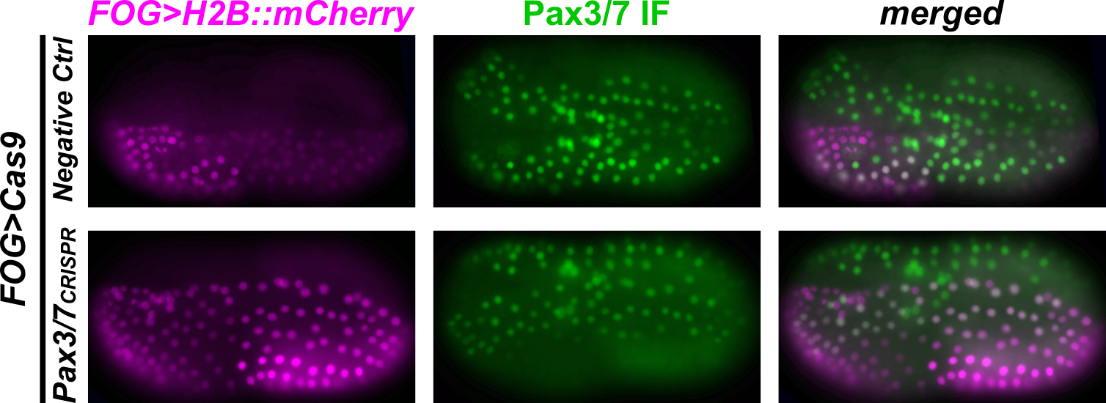
**

**Supplementary Figure 2. Pax3/7 IF in Negative Control embryos.**

Additional immunofluorescent staining of Stage 16 embryos electroporated with *FOG>H2B::mCherry*, *FOG>Cas9,* and negative control sgRNA. Left/right mosaicism does not affect Pax3/7 IF. Dorsolateral view in (b) shows IF staining is restricted to dorsolateral cells, as Pax3/7 is not expressed in lateral or ventral epidermis.
