# Supplementary material for: Pax3/7 regulates neural tube closure and patterning in a non-vertebrate chordate
Source: Front Cell Dev Biol. 2022 Sep 12;10:999511. doi: 10.3389/fcell.2022.999511 (PMC9511217; doi:10.3389/fcell.2022.999511)
Supplement: Supplementary file 3 [file Table5.DOCX]

**Supplementary Sequence File 1: Sequences used in this publication**

**Hypothetical cis-regulatory sequences (based on genome sequence, not validated by plasmid sequencing)**

ATG = start codon

>Pax3/7 -5877/+21

TGACATACAGTTTCTGATTGTCCTCATAATTTACTGTTAATTTGGATTTAATTTTATAAATCTCGTTACCGTTGTTTTGTACGGAAGTAAAATCGCAATTATTTTCACGGTCAAAGAATCCGTTCTTTCCTAATCAAGTGTCGTCCATGTCGTTAAGCAGAAACAGCTATCTACGGGCTCGGTCACCGGGAATGAATGCGAGGCGATAACTTTTCAAACTCGTCACTAATTTCAGTAAATCCAATAAAACGAGCGCTAAGAAGGTTCGGGGGTCGGTCGGCCGGGTCGCTGAGAGGTGACGCGAAAAGGACGACCCAGAAATCCAACAGACGCCTCGCAATACGTCACAGAGGCATTGTGTACGTCACTGTGATCATTGTTCGCGTTAGAGTGCTTGCGCGCCGTCACTTTACGCTCGGTTAAATTTACGTCGATTTTTTTTGCAATGATAGCGAAACGATTCGACGCGCACGCGCTGCTAATCAGCGCTTCGGTCACGGAAACGCTAAACACTCGTCAGCCACGCAACGAGGCGAGCCTAAAGAGTCCGGAAGAGCCGGCTTCGTTAACTCCTTTGTTTAAACGCGTTCGCTATTTCGAAAACGGAAGCCCCGACGACGTCGTTCGCGGGCCCAGCGTGTCGTCGAAGTAGCGAAGGTAGGTCGGGCGTACTACCATTTTAAAACAAGGTAGACGGATTTTCGAATGATAGAACTGATCGAAAATCGATTAAAGACTAGAATACGTCCGGCTGAGGCGATATTCGCACACAAAACCAACATTAAATCAGCGGGTGTTGTAGAAACCGAATAGAATCATAATCCGTTCACATAAATTGATTTTCCTTCCATATGGTTTAATAGCGTGGTCGTGCATTCGATTTTCGATTATGTGAACTTGCCTGGCGTTCCCACGCATGGAGTGTTGGTCGAAAATAAACGATTGTGACATCAGAATATGACAAAGCGTCAAGAAACAATAGAAAACGCGATTCCCACAATCTCCAGCTTCTGCGACGTTTTTAAAAATTCGTCATCTTAATTTCACGACGATTAACGAAACTTTGTTAAAGCGTTAGGGTTACTTTATAAGCTTAAACGCAACAGGAACAAACGAGAAATCCGTAAAACGGCTTCGAAACTCATCGGTGCGCAAACACATTAAGAGATTTAAATTTGCACCGAATATATTTTGTGACCCCAAGTTAACTAGGCGCCCGATCATCCTGTTACGTCGAAACCAGCCGAAGCTGGATCGAGGTTTGGGCCAATTTAAATGTCTGCTGCCACACAAAGAATGCTCGGTTGCAGTTTCCCATAAAACAGTGGTTTAATCGGGAACCCGCTGAGCCCGGGCGCCGTACGATCCTGTCAGTTTCGATCAGGCAGCACCCCTGGTTCGAAGGTCACCTGAAAACACGGAGCGGGGTCTCGCTTGGCCAGCCATGCCGAACGCTTAAGGAAACAAAATGATTATACAGCGAGAAGAAGCCATGTTGGCTCTGGACGTGGAGTTTGGAAACCAGGAATTGGTTTCACGCAAAGCTTTCACTTTTGTTGTCAATTAGACGCTCGCTGACTTGAGTAGCTCACCCGCTGCGAGATCGAGCTTCGAAACTCGACTGACAGGCGAACAAAATAATTATAAAGGCGTCACCAAAAATTCAATTGGGAAAGAACACGGAAAAGTTGAATTTGCGCATTTGGAAAATATAGAACTTTGCTTGTATTTTAAGAGCAGTTGCACTTTTAAGACCACATTATCGACCTGTATGATACATATGTTGGGAATAATGTCGATATTGCCAACTCTTAAAACGTTATATAACACCCTCATTTTAACCGCGCCAAACTCTTTTAAATTCCCACACCCGATTCGCGCTACGAACAACCATGACAATGATTATGATTGATTTACATCGCACATGGAGGTACAGAGATGACATACACGCTTGCTTGCCTTGGTTTATAACCATTGCTATTAGGTCGGGTAATGCGCAAGCCTTTCATCTAGCACTTGATAAGCTGTAGTTCTAATTAATTAATTATAAATACGTCAAATATGATGATTTTATAATCGAAACCGAAAACGGTTTCAAAAAAATGTCGAACTCCGCAGTAAATCAGTTAATACGAATATTTAGATAATAAAAGTAAAATATGTACAAAGTATCTATTAAAGTCACAAATACACACGGTATAACAATATTAATAAAATATATATACATACCTCTAATAATTTACAGTTAGCCTATACATCGTTTATTTGATTTTCAGAAAACTGACTCCTACAATATTTATCTAAAGATTTGCTTCCTATTTTTGCTTTATTTTACGTGTTGCTTTTTTGCTGTAACTTCCTATATTTATGAGGCAAGCATGTATGACTGTGTAAATCTGCTTCTGGCCGAACTGTGTCCTTTGGTTATCAAATCCGGTATTGATGCTCTCTTTTATGAATCATTCTGCGTCACTACAGATAACATAACATCAGTTACCTGCACCACACACAACAACTATAACACTGAAAACTGTGGTATCTTCTTTACCCTTATATTCTTCGGCGCCCTAACGCTATCGGATTTCAAATTTAAATTATCACGCGTCTTAGTTTCAACGTTTGCAAAATCACGGTATAGTGTTAAGACTTCAAAACACCTTCACAGTGTAGGTTGCGTGACCAATCGAGTCTTAGAAACCTCACGGCAGGTCTTCCACGGCGAATAAGTTATCTCAAATTTAGGTGCGAAAAGCTAAAGGGCTTATTTTCATAGGTGCCTCCATAGAACTGTGGCGAATGCATTGCTAGCATAGGGGCTTACACCAGCCAAACCATTAGCTTACCAATTAGGCTTATGGTTATCGCCAAAATCTCAGGGCCGATAATTTTCAGTTTAATTTTAGAGTTTTTTCTGAACTTGAATTTAAACGGTGCAGTTCCAGTAATAAAGTTGCCAGTTATTCCTAAGTTTTGTTTTAGTTACAACTTTTATTTTCTGCTTATTTGTATTTTTTTGTATAAAGTTTTTGGCATTTAAAAAGCCATTTCGTCGGTCTAAAAATTTCACACATCGTTCTATCTGTTCAAATAACAGAACACAAGCATTCTTTACTGCTTGATATACATTGTAACTTGAATTATAGCATTTTTCATCTCTGTTTTAACACTATAATGTTTTTAAGTGATTTCATCCAAAAATGGATTGTGGTTATAACAGATCTAGATAAAGTTAAGCCGGTTACTGGAATACTATGCTCGGTGCGCCATTTTTAGTGTATTTTGTTTGTTAAACCTCGGGTAAAATGTGTTCGGAGTGCCTTTATGCTTTTAAAGCGGGCCGTACATTATACAGCAACGATATTGTGTTTGGGGGAGAGTTTTAGAAAACCTCTTTCGTTCAATTGTAAGTATACCGTCCCTTCTACGTGTTGTTTGCACACGCTGAAATATTGCACCATTTTAACAAGCTTGACTGAAGTATAATTTTTAAACGCCGTTCAAATCGATAATTATACGCCGCGTCCTCCGAGACACCATTTTGTAGCAAGCGAGCCAACGGCGACCGGCCGGCCCGGGTCAGGTTGATGTCTTTCGCCGGAATTGACGACGCAAAGTACCGCAGGCGTGGGTGCCGAAGTTGTAAATTGGACTTTTCGGATTTTAGTTATTTTCCCAAATTTAAGTGAATTAGTTCAATACCTAAACTGCATGTAATTGCGCACATTGATAACTTTACACATGCTCGCTATTGTGTTATACTGTTTTGAAGACACATATTCTACACGCCTATCACCTATAAGCCTCTAATGCTATATAGATTGAATAATTTAAAAGGTGGTCCAAGGCAGCGGTATCTCAACACACAGTTTACTTCTTAATTGGAATTTAAAACTGCATTGCATGTCACTTTTCTGCTTTATTTCACGGTGTATGATTACCATATTTTGTAGCACGGCGTTAACGTTACGTGAGAGAAGCATAGATGTCGCACTACATCGGCCCGTGGCAACGTTTAGCCCAACACGTTTATTACCGTCGTCCATTTTTGAAAAGCGAAGGCCGATCCGAAATCGTGCAGAGTTGCAATCCTGCACGCGAATTCGCCTAATTATGTCGCTCGCCGTTAATCAAGCGTGGTTAGGGATAGAGAAAAAGATAGACTGGTGATATAGAGTGGCTATGACGACGAGCAAAGCGAGGCAACGGCAACTCAGACGAGCTCGGTAGTCTGGCGAGTCGCTGCTTTTTTCGGAGCTCGACCGCCGCGTCGTAATATCTTGCTCGACGACGCATCAAAACCAAGGCAATCTTATTGGCGTGTAGTGGTGGACGGAAACGGTCAGCGTGGTCCAGCCTTTCGTTATCGAATCGATTTTGGGCCAGAAAGCAAGTTTATGTAAAACCATGATATAGATGGGACTTAGAGTATTAAGGCATTCATCGGGGAGGCATTTTTAGGTGATTGGTTTTGATTTAAAGTCGAGTTATGATCCAAAGTATGATTACAAAACTAAAGGTTGAACTGAGCTGAAGCACCTTCACCGTCGTAACTTAAAAGAGATAAGCGGAGGGGACGCTGGAAAATTCAGTTTAAAATTAGATGAAGTTAAAGATAGGAAAAGAGGATTCGCTACGCCGTGGGTTTTTGCTTATTTTAAGGCATCGAGCTTGAAAAATTTATACAGTGCACCGTATAGTCAAGCCAAAGTTGCGTTACAGAAAAACTACATTGTTTAGCTTTGATCGGCCGGCAGCGTCCTTGCTGTTTGTTTGTTGATTTAAGCATTTGTGAGAATCTGTTGCGCCATTTTGAAATATAACAACCTTAACGAAAATTTATTCTAAAAGAATTTTCAAAAATATTTTGCCTAGTAGTTTATCTCTTACAAAACTACAAGCATCGATAATTACTTAAACTTTTCATAAAATACGCCGCAATATTTTCCAATCACAAAATTAGCTCCTTAATAAATATAAGGGTTATAGCTTTTATAACTCTATATAAACCAGAGCGTTATATTACTCGTTATACATATTGCTGTAAACTTCTGATAAATTCCAATTTTTCTGTCCTCCCGCTAAAACATGTTGTTCAATCTAAGCGTCACTTTTTCCTATCCGTTCTTTCAATGAGACCCGTGCCTGTAATTCGATATAGACGGTGGTTTTACTAAGACAGCATGACAGATAACTGATATTCTGGTTGGAGTTAGCGGCGAGAAAACTGTAGATGTCGGTTTTTAATGGCTTTTTGCGGAAGATAATTTCTAAATCACAGGGTAGTTAAAAATACTTAACTTACATAAGGGGTTTCTGCACAGTTTAGAATAGGGCCAATAATTACATAATTATGTAAACTTGTAGTATATTTTAAACTGTACTACATTCGTAGATGCGCCTAAATCAAGTATAAAAAATAAAATTGAAAAATTTACAGTCGTTTTGCGTTAAATACTTATTTTAAAAAACGCCAGGTGAGCGTTGCATGCGGACGCCATTTTAATTTTACCTTTCAGATATCTCATGCACTTAGCTTATTTTTCGTTTAAAATCAATTATTTTTAACCGATTACGGTTTAATGTACTAGGGTATTAATATATTTATTATTGTTATGTGGTATATAGTATAGTAGCATTTTTGTCAAAAATGAAATAAATTCAGTCGCGTTTTAGATGTATTTGAAATGTTGTAAATTTTATTTATATGTTTGAGAAAAAAGAGTGTAATTAAGAGATGGAATTCGTTTTATTTTTGAACGCTTTCATTATTTTGCAGGCCCCGAATTGCTTTACAATTGCAATAAATCTTATAAGAGAGAAAGATTAAATTATTGTTGTAAAGTAGTCTGACTTCTGAACACAACCCAACAAACATGATGCATCCAGGGTCTAATTTCCGA

>Pax3/7 -5877/-4121

TGACATACAGTTTCTGATTGTCCTCATAATTTACTGTTAATTTGGATTTAATTTTATAAATCTCGTTACCGTTGTTTTGTACGGAAGTAAAATCGCAATTATTTTCACGGTCAAAGAATCCGTTCTTTCCTAATCAAGTGTCGTCCATGTCGTTAAGCAGAAACAGCTATCTACGGGCTCGGTCACCGGGAATGAATGCGAGGCGATAACTTTTCAAACTCGTCACTAATTTCAGTAAATCCAATAAAACGAGCGCTAAGAAGGTTCGGGGGTCGGTCGGCCGGGTCGCTGAGAGGTGACGCGAAAAGGACGACCCAGAAATCCAACAGACGCCTCGCAATACGTCACAGAGGCATTGTGTACGTCACTGTGATCATTGTTCGCGTTAGAGTGCTTGCGCGCCGTCACTTTACGCTCGGTTAAATTTACGTCGATTTTTTTTGCAATGATAGCGAAACGATTCGACGCGCACGCGCTGCTAATCAGCGCTTCGGTCACGGAAACGCTAAACACTCGTCAGCCACGCAACGAGGCGAGCCTAAAGAGTCCGGAAGAGCCGGCTTCGTTAACTCCTTTGTTTAAACGCGTTCGCTATTTCGAAAACGGAAGCCCCGACGACGTCGTTCGCGGGCCCAGCGTGTCGTCGAAGTAGCGAAGGTAGGTCGGGCGTACTACCATTTTAAAACAAGGTAGACGGATTTTCGAATGATAGAACTGATCGAAAATCGATTAAAGACTAGAATACGTCCGGCTGAGGCGATATTCGCACACAAAACCAACATTAAATCAGCGGGTGTTGTAGAAACCGAATAGAATCATAATCCGTTCACATAAATTGATTTTCCTTCCATATGGTTTAATAGCGTGGTCGTGCATTCGATTTTCGATTATGTGAACTTGCCTGGCGTTCCCACGCATGGAGTGTTGGTCGAAAATAAACGATTGTGACATCAGAATATGACAAAGCGTCAAGAAACAATAGAAAACGCGATTCCCACAATCTCCAGCTTCTGCGACGTTTTTAAAAATTCGTCATCTTAATTTCACGACGATTAACGAAACTTTGTTAAAGCGTTAGGGTTACTTTATAAGCTTAAACGCAACAGGAACAAACGAGAAATCCGTAAAACGGCTTCGAAACTCATCGGTGCGCAAACACATTAAGAGATTTAAATTTGCACCGAATATATTTTGTGACCCCAAGTTAACTAGGCGCCCGATCATCCTGTTACGTCGAAACCAGCCGAAGCTGGATCGAGGTTTGGGCCAATTTAAATGTCTGCTGCCACACAAAGAATGCTCGGTTGCAGTTTCCCATAAAACAGTGGTTTAATCGGGAACCCGCTGAGCCCGGGCGCCGTACGATCCTGTCAGTTTCGATCAGGCAGCACCCCTGGTTCGAAGGTCACCTGAAAACACGGAGCGGGGTCTCGCTTGGCCAGCCATGCCGAACGCTTAAGGAAACAAAATGATTATACAGCGAGAAGAAGCCATGTTGGCTCTGGACGTGGAGTTTGGAAACCAGGAATTGGTTTCACGCAAAGCTTTCACTTTTGTTGTCAATTAGACGCTCGCTGACTTGAGTAGCTCACCCGCTGCGAGATCGAGCTTCGAAACTCGACTGACAGGCGAACAAAATAATTATAAAGGCGTCACCAAAAATTCAATTGGGAAAGAACACGGAAAAGTTGAATTTGCGCATTTGGAAAATATAGAACTTTGCTTGTATTTTAAGAGCAGTTGCACTTTTAAGACCA

>Pax3/7 intron1.1

GTCGCTAACTTAGCAAGTCGATAAAACACTTGCTACCGATGCATTATTTATAAAAAGGTATCGAACCCAGTTTATAGACTTGCGGTTTAACCAGTAAGCTACGATCGACCGGTTCTTATCGACTACTGATGTTAATCAAACTGTAAACCGAAGCCCTCTCTTCACTAGTTAGCTTAAACCGCCTTGAAATTGATCTAATGAAATAACTACTTTGTTAAACTTTCGCTTTAAAATTGCCCAAACCTGATTTTAAGTTTCAGTGTTATTTCTGTAGGATTTAAAATGAATTGGGTAATGAAAGTAAGTTTTACAGAAAGTGTGAAGTTTAAAAATTAAACTCTTGTCCGAATCAAGTTATCACGCCTCGGAATCCGATTAACGGAGAATTACGCTTTGTCTAATCGTACAAATCCTACACACGAATAACTTGCTGGTCAAGTTGCTGACCCGTGTTCGCGGCTGGTGCATAATTACACAGCAACTCGTGTTCCCACGAGCAAATTTAGCCATCAATGCGTCGAAGTGGTGTGAAAATGCAATCCGCAATCGAAGGTCATTAAGTTGTGATTATGATTTTGTTTTAATGATCGCATGCTCGCTGCCGAGCGCAGTCGGAACGAACGACAATGAGTGTTTAATTATGCCCAGTATTGTCCCGGGCGTATCGCAACAATGGCTAGCATCCAGACCTTACCGTCTCGCATGTAGTGCACGGACGATGTTGAAAATAATAGGGAACGTGTCTATGCAGTTTCGACGGCCGTGTGGGAAAACAGATCACAAAAGATTGGCACAAGAGACTCCGGTTCGCCGGAAGATCGGCATTACACCTGTTATCAGACGTTCGCAATGAATTTTTAACTTCATATCTTATTCTACTTGAGATATCCAATGCAGCATCAAATTCAACCGAAAAGCTTGAATTTTAAAGCAGCATATTCTAAAAAATATCGATTTAAAACGAGATATAAATTCATATCGAATGTTGGTATAATAAATAACACGGAACATAAAACGACTTCTCGCCTTTCTGTTTACTTTTCGAGGTGTGTAATACGCTGACAACTTTGACGCTTTCGGCAAAAATATTTGACCGCGAATGTAAACAAATCGCTCCGACCATTGTCCTTCCTTGCAACGAAAACGCAACAGTGACGCATTCTGTCGCAATGCTGCCTTGAATTGGTCACCTGTGTTTACAAGCATGATGGCAGCTGGTATAAGCGGTGTGATAATCGGGAAGTTTTAATAGGATTTACCTGCGATGCGGTATCGTAGATGACAGGACGCCGCCCATACAACGTGTCCGTGTATTGGATTCTCGCCAATCGTTGTTGCGGAGATGGCATGGGAAAATAAACCGCATTGCGTGTGTATATCGAGCATATATAATGCCTTATAATAGTTTATAGGGGTTTGCTGGAAAATTAAGTGTTTTTTTTTTATTTCATGCTATTTTATACATAATGTAATAAAAATACTTATTGTGTCCATATTTTGAAATGGCACACATTGCTGCATGTTGCTTTTTCGC

>Pax3/7 intron4.1

AACATGTCGCAAAATTGGTCACTTCCTTCCGTGACTGTATAATGTCGCCGACCCCAAATGACTGACATTTATAATCTAACCGGTTGTGAGAATTTAAATAGTTAAATCGAACAGGTGAATAAACATCATCGCAATTGAAACTGTTGCTTCTATGCAGCAAATACCTTGCCGAAATAAATAAGCGCCAACTGGCGGGGAAATTGCCCCACTACCGTGTTTGCACAGTCACTATAAGCCGCTTAAAGTGTCAAATGAAAACACAGCCGCAGCCCCAGTAGAAAGAAAGTCCCCATAATTTCACTATCCTAACCACACTACTTAGTAGTGGTCCACGGGAATAGACGAATTTGGTAATTTACGTGGCCGAGCCTATACTGGAACGTGTATATCTCCGATTCTATTTACACAGTGAGCATAAAATTGCTTAACATCGAATCAGACCGGGTGCTTTTGAACCTGGTCTAAGAAAATCCTTTGCATTCAAATGTGGTTTATTGCCTTTAATTGCGTTATCAACTTGTATACAACTCCTTGACGAGCTTAATTTCCCGAAAGCTTTACTTCGCATTGGTAATCTCGAATTGAAATGCAGCATTTCGACCAGATATTATCGGATATCAATTAATTCCGTTTCCACGGAGACAATTAGCCGTTGCTGCTTGGTTGTTTTTGCGGCTGAGCGTTCGGTACAGCATATGGGAGCGGTTTAGGGACCGCCTCACGGTCAATTTTACGCCGCTGTTCCGCCCCTCCTGTCGCTGTTGCTCGCCAGCGGCATAGCCTGTCTCTTCACCGCCTTTTCTGTTCATTAAATTTCCACCGTTAATTCGCTGCCGTTAAATGGCCCGTCGACGTTCGCCTAATAGACAAACCTGACCGTGATAGTGAAACATGCTGTTATGGCTTTAAGATGAGGCTGCGATAATGGCTGTAATTTAAATCATCAACCGGAGCTTTTAGAGCCGTGCAGAGCAACACATTAACGAAGTATATTATACCACCGTTATCAGCAAGTGAAACTAAACCTAGTAAAAATGGCGCTTTGACTAAATGTCAATCATCCGTGTCAAATGTAAGTTATTAAGTGACAATAAATGCGGGGAAAAGATACTTTGTATCTTCATTAACTGCAATAATTAAGTGGAACGGCTACGAAAATTGGCAAGCGTATTTCACACACAGTCTAAACGTCCAGCAGGGCAGGTGTATTTAACATTCGCCGCCGGCAATTCCCGCCATTTTGTTGGACACCTGGACAATAGAAAAATGTCCAACCCTTAACACCTATACAGCGTGTACTCGGAAACTGGTGGATGTTCACATGCTTTAAATCGCCGGGTTGATTTTCTAAGTATGTGCGCTACGGAAGCCGGGGAAAACGGGATAAATTAAAGCTAAGGGACACCGCTGCGCTAACACTAAAGCTGGAGTTAAGTGTCGATTTGAGGCATTAAAATAGACGCATAAGGGTCGGAACAATATAGGATTTGAGTCCTGATCGGGATTTAGACGTTCACTTTAGCCCAAAACAGATGTCGCCGATTAGGAACTGTTACCAGCGTGATGTCGTGTCGAACACAATGCCTTCAAAGCAACAGTACCAACGCTTAAGTGTCAGCAGCGCCCAAATAGAGCGACAAATTGACATAGCGGAGGCGCTATGCCTTAACCAAGTCACAAAGTACGAAAACCCTTCCCGCCTACTGTGCTACAACAGTTTCTGGAATTTCGACTGCTGGTTATTATTGGTTGGTCTTCTTCCTGTGTTACGTAACAATACCTCATTCATCGAGGGCCACCCGATTGGAATATCAAAATGACGTCATCACTGTACAGTCACTTTAACAGTCTAACACGATAATAACCGACTAAATCGGTTATGTTCATCCGCCTGCCCTACCCTGTCCCATCCCTTCACCGTGTTGGGTTGCGTTAATTCTTTGATACTTGTACGAGTGTCGCGAACTCTCCACACAATTATTTTGTTTGATTATTAATAATAGA

>Fgf8/17/18 -4833/+12 (Imai et al. 2009)

GCATAGATTTAAGCCACCTCGTGAAGTGGGCGGGTTTAATTAAAACAAACAGAGCTCCAGCAATCAGTGCAATCAAGACGTTTGTCGTAGGTTAAAATATTGCTGCTAAAGCCGCTTCTGATCAGATAAATTATGATAAAATCAAACCGCATTTGATAACATCTGCTCCGTATTGTTTTGCCGGTAAGTATGGACATAGACATTAAAAAATGTGGAATAAATATGAAAAACGCGGTATAATGATGGACTATAGGTATATGCCTGGTTTGAACCGAAAATGCAAAGGTCACAGATCTAAAGCTGTTGAACGCAAGGTGTTCAAGCGAAAATTTCTGTGAAACTGCTGTTTGGTATTTTTCCGATGCTAATTCGGTGCGTTGAGAGAACCCCATGGAATGCTGTAATATGCTAGCGTTTGTTGTTACAATAAACATACCTAAGTTTAAGCGTTATAAAAAGCAATACACCAAACGTACATGTACAGAAAAGTCGTGGTTTACCAATAATGAATAAGTTATGTGTATATATATAGCACCGGTATTCTATTCTTATCTCGCAACCTCAGTTTACTGGACTGAAAACAAATCGGGCTTGTCTCCCTTTGTTCTGAGTCTTGTCCACAACAAAGCGTCAGATTGCGCTGGTTTAATGATCTCGAATTTCAGATCATCAGTTTTCAGTTTCCCAAATATTCCAACTATCCAGTACCGCTAGCACAGGGTACACAAATAGGCTGTGTATCGGCTCTAGCGACTTGACCTCGTCGGTGTTTGTGCAAATTTATAATCCCGCCAGCCTAAACAGGGCAAGGGCCGCTGAGGCCGCATTGACATACAAGTACGCGGTGCTTGCAAAATATCAGTCCGCAATTTCTCGCAAAAAAACGTGACTGCACTCTCGGGGTAGCGCATTGTTCCGGCGGCTCCTGCCTGCACGGCGTTTTCATGAATCACGCGGCTGTCTAATCTACGATCAACGGCCTTAGCCAAAAACCCGGATCGGTAAAAGTTAACACTGTTGCGAACTCAATACCTCGAAATTCTTGAGGTTACTGCGGTTACCCGGGCCGGATTATCCGTCAAAAATAAGCGAAGTCGGGTTCAGTTTACGTTTCTCCTAACTATTCCCAACTAAAAATGACAATTAAAAAAGGTATCATGATATTGTGATTAGCATGTCATCCAGCAACGAGCTGTGTATATGCAGGAGCATGTTTTCCGAAAGGATTACTATTAATTTTGAAGTTTGATTTCAGAAAATCGATTATGAGCAAACAAGTAATGTGCGGAAAAGATTCGTAATACCTTTTTACGAAGCAGTGTAGCAGTTAAAACAGTAAATCATATTCTGTTACTAAAGCAGTCGATGTTTAAACGGCTGGGTGTATCTCGCTTTCAAGAGTCGGCGTCGGTGTCCGGAAACCCCGCTTCTCCCAGAGGTAGTGTTGTGAAAAATGCCGGCCCAGCTATTCTCATTGTTTAGCCTCCGATAATGGGAAATACAACGCGTGTCTGAGATACCGCTTTCCCATTCCTCAAGTACGTTGTTCGCCTGCCTGCCGTCCATTGACTGCTGGTTAATTACATCAACTTGTTTTATAGGAAACGCTTATCGCACACAGAGAGCGTGACGCACAGAATCAGAGAGCACATGTAATATAAAGTTGTATGTAAACAAGGGAGTTTTAGTCCTTAGTGAGCGCCATACGATTGCGATCGGATGTTTATCAGCCTCGGCTAGTGCACAGTTTAAACGTTCTGATGTTAGTTGCCGAGCCCTCAGCAAGTATGTATACAGCTGTTTATTTGGGCCTCTGTAGTTCTAGTGTTAAGGAAGGCAGACTCGACTGCACGCTACAAGCGACAGCGCTTTTTCCATCAATCCAATCACATTTGTTGAGCCCCTTACGGGCACTTGAGGTGACGGCGCTAATTTCTAAGCAGTTTCGATTGTGTGGGCAGCTTGGCCACAGAGCAAAGCGCAGAAAACGCGGCGCTTCGCATTGCAGCAGCGCCGAACACTTTGCCAAGGTTGCAGTATATGAATACAGTAGCGCCGCGAAAGACGGAGAGTTTTTAAAGCACAATAAGCGTGCCTGCGGTCTGTACATACATACAGCAAAATTTTCGGCGTTGTGTAAAGTTGGTCGCGACAACAATAGCCCTCTCGGTGTTTGGTGCTTGAAGCGGCCCTACAACAGCCCGCTATCATAGGCCGGGAATTAGGGCAAGTGGCGGCTTAGCGAGAATTTAATTTTCCTTGTTAAGCAAAATCGTGTGAAGTGCTTCTGTCCCACGACTGTTCACTTCCTGCGACAACGTATAGTGAGATTGTACTTTTGTAGCCAAGATACCAATATCAATAATAATATTCAGCTTTGCCCGCTAGGTTGGAGTAAAGGGTAGATATTAGTTGAGAATAGTTGCGTAATATGATTAAGCTCGTGTACCGGCTTGCTTTATATATTTTTGCTAAACAAACAAGTGTAGCGATTAAAATCACTCAGGCCTAAGCCAAGGGTAAAACCGGTTAATTCGGAGGTTTTAGAAACAGTTATTTTTTGCGGTTTCACTAAACTTAGAATAAATACCCAAGAATATTATTATAGTTTATAATTTTTTGTTTAAGTTTTTTTTTTTAAATATCCATTTTTTGGTGATTGTGATTGATTTGTAATAAGATATTTTTAACTTCGATATTAATGTTTGCTTAAAATTACGAAATAGAGCCGTCTGCTGCGAAGGTTTGTAAAGTGAAACAATTAGTGGGCTACTGCAGTTCTACTTTCTGTACAAATATACGCTTTATCACGCTGTCACCTTGACAGACGTTATTGTAGGAAAGTTTTTGTGCTGAACAATAGCCTTGTAATTAGTTGCGGGGGGGCCTCTCGGTTCAAGGCCGAATAGATACGACCTCTGATCAGCGGGTCGAAAACGAACGTAACAATAGGGAATTATTTCGTGCTGTGACGTCGGCCGAGTGGGGCTGGGAACCATTTCTAAAACATTCCTGGCATAATGATACGATGTAAGCGCAATATTTCACAAACGAAACAAGTTTACGTGACTGTAACTGCATAAGCGTTGCTTTGTCACTGGAGGCGCCAACCGCACGATAAGATATTTCAGCGACGGTCGCAATGAAAACGGACACCCTATTACATGTATGACGGCCGTTAATACCTCTGTCGCTTTGACACCTTCCAATTGTCGATAACATAACCCGCAACTTGATATTCTGTGAATATTATGACACCCGGCGCCTTTTGCCTGAATTGTGTAATCGTGCCGCAATATTCGAAATTGTCCTGGGTTTATTTTTAAAGGCAGACGTCAGAGAAATTGTAATTCTTTACATCAGATCTGATAAACCGCTCGATTTCACGGATGTGTTGAACAAGCGCCGTTTCAAACTGTCGCAGTTTTATTTTTATCTTCGTATAAATTTACAGCGGAATTCATTGAATGCTAAACTGTTGTATAAAGAAGTGTGCGGAAAGACGGGCCCTGTTGGGACATACTATCAAACTATTCTGATCGTGTTTTAAACAATTAACAACGGTATATGGGATTCGCGAGAAAACGGTCTTATAATTTTTGAATATTCTTTGTTTACTACCGAATAGGACAAGAAAATAGAATGAATAGGTGTCCCATCTTTCCCCACCCTATACTATATATGAACCACACGTACTCCAAACACACTTAACAGCGTGACATATTTCGCTATTGCGCCAGTTTAAAACACTTTTATCTTGAGTTCAATAGGCATACGTGTCATAGTTACTACACACGTTGTAACTTTTCCCGTATCGGAAGTTTTTTTTAAAAATTAGGACTCTCCTATGACGAATTATATTAATTGCTCCCCTGGCGCGGTTTATTTTGTCTTTTCCCACTTATTTTCCTTTGCCCACGTTTTATACAGTTACCTTTAATTTGCTACGCGTTTGCTCGACGGGCGGCATGAGCGTTTTGGGGATTATGACGTAACAAAACGGTTTTTAATCTCTTCCTCTATGTTACGACATAATAGCACCTGTGGAAAAACACAAACCTGTTTTGTTGGGAAGCTGTATGGTTGTAAAGGGATGTGTGTTTCGTTTTGTTGGTCTCTGCTAAATACACGAGTGCAATTTCAGTCAATTACGAATACCAACGTATGAAAAGAATGCGCAGATTTTGCTAATTGGTTTGTTTACCCTCGTTCGTGCTTGGAATTCTGCAAATTGTTGTTGTGTGGGTTAGAACAATCCAATCACCCTGAAAGTTTTTTTAAATGCAGAAGAAAACCATTAATTTTGTGCCCAAAACACCGTAAGCTGTAGCCAGCACAAGTTCGATATGTGTTCATCAAAAATCGGCGGACTTTTTCTCACCGTCGCTTTTTTCCCGCGGCTCCAGCAACGCGTTTGTTAAAAGCGCGTTGCCTACCGTTATTTACATTAAACGATGCCGATCGCTGGGCGGTTCGTTATTTTTACTCGTCTGGTAATGGACAAAATAACAACCCAGTCGTGTGCTTAGATTTTAGCGGTATGGAGCGCCACGCGCCGACCCTCGGGAAGGACATGCTGCACAGCGGGAAACCGAGCTTTAGGATCCGGCAGATAATTTTATTCGGAGTCGATGTTAGAATTGTTATAAGTTGTTCAGGATAATAGCAAAGTGAAAGCAGAAATAAATTTAAACTTTATGCTTTGTATATTTATTCAGTAAGTAGTTAAATAATGTCAATTCGGATTTAATGCATTGCGAGTATAAATAGTAAATCCGAATATCAAAGTGATTTCTAAGGGACATTATTCTTCTCTTGGATTACATACGAAAATGCGACCCTCC

>Dmbx -3490/-2159

CACTCATCTGCCTACATTAGCTTAGTATTTTAGTTGAATAAATAAGTTCCAATATTTTCACAACCCGTTCTTAACTATAGAACAAGAATCGCTACGGCTCCGATGCTCGATTTCGAGTACCCGGTCGCCCTAGCAACCCACTTCACAATAGTAACAATGAGACCCGACCCTCCCATTGTGAGGTAATGTTTCTTTCATTGTTACGTGATATCGAAACTCCGCCAATCGCAAGGGAAAGAAAATCGACAGGGCACAAAATGAAATTTTTCGAAGAGTTAAAAACGATAAAAAAACAAACTAAAAATACTAAAAGTTGTTGATTGTTATAAAAACGCGACAGAAGCGTTTATTGTTTTATCACAATGACGATTAACCGGACCATACTATGCCATTGAAACCGCCTAATTGAAATTTCGTGGTCGGATTCGCTATTTTCGCACCCTGCGTTCCTCAATTTGATTGTATTCTACATCGTTAATATTTAATGAAATATTGACTTGTTAATCGACACTTCGCGAAAGAACAATTAAATCGTTATGACGTCATATGGGTAGACGATTGTTTTAATCGCGATGTTTCGACACCACCTGTTAAAATGTTTAACGAAAGAAAATACTAAATTAAACTTTAGTACTTTAAACGGCAATTGTTTCAACTTCGTATTCACTTACGTCTTAGGAGTTGTCTAATTTGTCAGTCATACAAAGGGGTAAAGCCAAAAAATAATCACTCACATAGTTACATACATATGGTAACTCGTAAGCTGGCACGAGGTGTATGAAACAGAACACTCGTGTTATATACGACGTTGGTCATAACGACTGTCGTTTTCCGGCCACGCGAGGATAAATAAGTTGCATTTACTCGGCCCATTTAGTATAAAGAAGTAAAAAACGTTTTTATAATAAGCAAAAACTAATCAATTAAAAGAAGTTTTTAAACAGATCAGGTTGATCGTATTTAACCATGGATAAGAATACAATTCAGCCACATAAAGTTTCGAATTTAAAGTTGATTTATTTTAATTTATTCGAATTAACAAGCAGTCGCCGATAAATCGAAGACACATTTGTGACGTCACGCCTTAACAATCCGCTATTGATGACGTAATAAAGGTGAATCATCTGCCATTATGAGCTCACAATTAACGCGTCTGATCTGCGACAAACGAAGCGCGAAATTGCTCGACGCGCTGGGAATAATTAACCTCGCTTTAACGCCATCTAGCGAGCACGAATTCCTGTATATTTAGGAGCGTAAATAACCAGAATACTCATTCGTGGTTAACAATCAGCATTGTTACGTCAATAAGGATTGTAACGTCATAGTACT

>Eef1a -1955/-1 (Sasakura et al. 2010)

GTGACGGGAAAACGATAGTCGTTATAACACGAGTATTCGTACACCTCGTGCGAGCTAACGAGCTACCATATATGTTGTGGGCGAATAAAGGTTTTATAAATATAACATTGGTTTTATAAATAAAACAACGCCATTTTAAAGTCGGTTACATAATTCTGTAACTAGTTCAAATTGAACGGTAAACGTAAATAAAAACCTTGACCGTCTTACCCAATTATATAAAAACACTTTGAACGCTTTTTAAGATGGAAGGGTATGGCCATGCCTAGATAATTCTGTGGACCATCTCACCCCAACCTATTACAGAACGGTCGTAATAATGAAAATGGGTACCATTTTTAGGCATATAGACTGATTCCTCCTTTCTAGAAACGTAAGCAGTATACACAGAAAAAATGAAGTGTGATTCTGTGCAATTAAACCGTTCTAAATTCATAGCCGACTGAATTTCTAATTAAGTGAATGTCTGACCTAGATTTATTGTTAAGTTTAGCACCAAATCTGAGCCAGCGATAAGCAGTCTAATTAAATTGGCTGCTGGCGATAAAATAGGTCATCCTGAAAAATCGTTTGCGCCTTTATTTAAAATATAGTAGAGTGGGGAAAGACGGGACATCTTATCGTTCTATTTTCTCGTCCCATTTCGTAGTAAACAAAGAACATTCAAAAAATATAAAACCATAACTTCAAAACTTCAATAGACCGTTGTCAACTGTTTAAAACACAATAAGAGAATTTGGATATTATGTGCTAAAGGTGTCCCATCTCCCCCCACCCTACTATATCTGTTTATAGTTCTGTGGGGTAAGATGAGATACCGTTAACACCTAAACATTTTTACTTTAAACAATCAACCACGTTTTTTATAGTCGTAATGGACATGTGGTTACATAATTCTGAAAATATTTTTTGCCCCCGACCAAAAGACGCGAAGAGTAAAAACATGTCTCAGCTTATATTCCCCACATAAATATATTTTTGTACTGTTTGGTGAATTTATAAACTTATATTACCATGCATATACGTTATGTTACTGGTATTTTCTCAGTAGGCAAATTCATTTGTCCACGTTTTATAGGTTTTCAATATTTATGATTTTTAAAATGCTAAAAATGTGGGAGGGGGGTTGAAAGTACAATACAAACACACAAAACAACTCAAACTAAAGATTTATAGTTATGCTAATTCACCTACACAATATAACAAGATGTGTAATGCAACCATGTGTTTATGATGAGCGCTAACATATTTTGTAACCACTCAAATTCCCCGCCACACGAGGATAATGAATAGGTGACTCTGTAGTCTGTACATCTTAGACTGAAATAAAGATTATAAATCTACGAAATAAAATAATTTCTGCTCACTGATTATACTTCTGTTTTATAGATTAGAAACCGTTTCTAATAAATGACCTAATTCGCTATACACACACGCTGTGCGCGAGATAATCATTCTCGCACCCCGTTTATTGTGTTAAAATTGCCGCCTAGATTCACAAAGCGTGACGGCTAGAGCCAGCAACGTGTCGCCTTCAATTACGCAACATCCGGGTTGCGCAATTCTGGATATAAAAGAACTAACAAAGATGACGTAGCTACCTTTTTCAGTTCAGACTTACGAAAGACTCACGTGTCGGCGGTCTACTTGTCCTTTTCGAGCTGTGGCAATTTGGTGAGTGGTTCTATCTTATATCTGAGTACATCTCTAAGGAATTATAGTTTGATTAGTTAAGTTTTTATTGTTAGGAAAGATGAAATCATTAGGTTTTACTTAGTTTAAGTATGTTAGTACTGGTTAGGCGTTTGAATTATTGAAAAACTCAGTTCGTTAACTGTAGTAGTTCTGGTAGCTTAGCAAGTATACCCTGTATACGCCTTTTGGCTTTTTAACAATAACTTAAACTTATTTTACAGCAAATTTCTGTGCATTCGGTTAACCCCAACCTTCCAAA

>FOG -1982/-1 (Rothbächer et al. 2007)

GCAACTATTGTAACACCACACGGGGGCGCAGTAGAGCCAGAATCTTAGTAAATGCGGTTTGATTGTAAAGTTTTTAACAATCTCTCGCTTTGTATATTCTAGAATGGGGAAAGATGGGACCCTTAAGCACTCATTGCCCAATATTTCCAAACCCAAAATCTACGATGGTTTTGTGTGGTACCACGAATTAGTACTATCATATACACTTCAAAGAAATATATTAAACCATACGAGAAGTAATTTATCTATTCGCACAACAGGTGTAACTTTGGAAATTCGAACACAGGCGAGCTAGTTTTATGACACGTAAACTTTATATATTTCTTTTCTAATTTAAGGAATATTGACCACTGCCCATTTTTCTTGTTTTAATATCCCAGTTTTTAAAATGGCCTGAAATATTTCAGCTTATTTTATTAAATTACGATACTGGATTGATGATTCAAATTAACAAACTACCAAAAAATGTTGACAGTTTTAATATGATTTACCGAGCACCACGTGTAACAGCATCATACAAATTTAATTGATTTTGTATTACTTACTTTATAATTTAAACATACGGTATTTAGTAAACAAAGAACATTCAAAGAATTAGAAAACCGTATTCCCATGACTTTCATAGACCGTTGTTGATTGTTTAAAACATGATCATAATATGTAGGTATTATATGTGCCAAAGGTGTCCCGTTTTCCCCCACCCATACTATATATATTATAAAAAAAAATGGCGTTATAAAAAAACTATACCCTGCATGTATTTATAGATTTGTTAAACGTATGATCTCGTGTTATGATCTATCCGTATAGTGGACCAGGAATCTTTGGGTTGTGCATATTTAACTGAGAAATCGACAAGCAATTAAACGCGGCAGTATACGACATGAACAATATAATTGGCGCATTATAATTAGATTGTATACCACGCACTACCTAAAAAGTTAAGCTATAACTGACGTCAGGTATTATTTATTGGTATCCGGCAACACTAATAAATGAAATTTTGTCAAAATGTAAGCCCTCGTATCTATTTAAATTTTGACGCTAAATAATCCATTTATGCAGTATAATACCAAACATACAAAATGTTTCAGTTTGTATCACTTTATTATGCATCACGTTTTTGGGATAAATACACATCAGGATTTAACAAAATATGTTAAATACAATATGGCGGTATTGCTTTCACTTAAAAGTGTTAACAATTAAGATGGGAAAAATAAAACAGTAAATATATTCTTTAAATCGAACTTTTGTTGTAAATCTTCTATTTTAATAATTTATTCATGTTTAAAACGATGTTTGTTTCGGCGTCATGCTGCTCGTTAGGTATAACTTCACTCGGGACATTTTAGTATCGAGTTGCTCAAAATTGTTAGGGTTGTCAACTGAATAGCGATGTTTTCGATTGAGTTAATATTTCTATACATGATGGCATCAGCTAGTAAGATAGAAAACAACCTTGTTATTACTCGTGCTGTAATAATATAATTCATGAAAAACAAACATGGTCTTATCAAATCTATTCCGAAGACAAACATACGGCTGATAGAATTCAGTCAGCGCGGTTTGGGCGACATCACTCAGAGCTCTAGCTCTTATCTTGCTTATCGAGCAAGCGATAAAACAAGCAGTAACGAGAAAACAAGAGAAAGAAGGCTAGCTTCCTGGAGAAGACCAAGATAAAGTATCTCAAAATTCAGGAAACGGTCCAAGACCGAAGCTCCAAAGCTCTTGTGTTCAGTTAAACTCTGATAGTGAATAAGCTTCGTGTATTGTACCGACCCATTGTCAATCATGCAAACTTGATATTATATTGACAAGAGAAGAAGGCAGTTTAAATTAAAACTCTAAAGTAGAGAGACATTAATCTCAGCTGACAAGGCAGGTGGTCACAGTAAGTTCATTTAAATAGTTGGCCAACAACAGCTTTTCCAAGAAAGTATTTTTGTTTCAGGTCTATACAAAAATAACACACATA

>bpFOG (Rothbächer et al. 2007)

AGCTTCGTGTATTGTACCGGCCCATTGTCAATCATGCAAACTTGATATTATATTGACAAGAGAAGAAGGCAGTTTAAATTAAAACTCTAAAGTAGAGAGACATTAATCTCAGCTGACAAGGCAGGTGGTCACAGTAAGTTCATTTAAATAGTTGGCCAACAATAGCCTTTCCAAGAAAGTATTTTTGTTCCAGGTCTATACAAAAATAACACACAACATG

>H2B(Helobdella leech)::mCherry (Gline et al. 2009)

ATGCCACCAAAGCCTGCCAGCAAGGGAGCTAAGAAGGCCGCCAGCAAGGCGAAAGCTGCTCGCAGCACGGACAAGAAGCACAAGAGAAGGCGAAAGGAAAGCTACTTTATATACATATACAAAGTGCTGAAGCAGGTTCACCCGGACACGGGCATCAGCGGCAAAGCCATGTCAATAATGAACTCGTTCGTCAATGACATCTTTGAACGAATCGCAGCCGAAGCTTCTCGCCTCGCCCACTACAACAAGAGATCCACAATCACCAGCAGAGAAATCCAGACGGCCGTCAGGCTTCTGTTACCCGGCGAGTTGGCCAAGCACGCCGTCAGCGAAGGCACCAAGGCCGTCACCAAGTACACCAGCTCAAAGGTCGACAGGCCAATCTGGCCGCGGGTCGACGGTACCGCGGGCCCGGGATCCATCGCCACCATGGTGAGCAAGGGCGAGGAGGATAACATGGCCATCATCAAGGAGTTCATGCGCTTCAAGGTGCACATGGAGGGCTCCGTGAACGGCCACGAGTTCGAGATCGAGGGCGAGGGCGAGGGCCGCCCCTACGAGGGCACCCAGACCGCCAAGCTGAAGGTGACCAAGGGTGGCCCCCTGCCCTTCGCCTGGGACATCCTGTCCCCTCAGTTCATGTACGGCTCCAAGGCCTACGTGAAGCACCCCGCCGACATCCCCGACTACTTGAAGCTGTCCTTCCCCGAGGGCTTCAAGTGGGAGCGCGTGATGAACTTCGAGGACGGCGGCGTGGTGACCGTGACCCAGGACTCCTCCCTGCAGGACGGCGAGTTCATCTACAAGGTGAAGCTGCGCGGCACCAACTTCCCCTCCGACGGCCCCGTAATGCAGAAGAAGACCATGGGCTGGGAGGCCTCCTCCGAGCGGATGTACCCCGAGGACGGCGCCCTGAAGGGCGAGATCAAGCAGAGGCTGAAGCTGAAGGACGGCGGCCACTACGACGCTGAGGTCAAGACCACCTACAAGGCCAAGAAGCCCGTGCAGCTGCCCGGCGCCTACAACGTCAACATCAAGTTGGACATCACCTCCCACAACGAGGACTACACCATCGTGGAACAGTACGAACGCGCCGAGGGCCGCCACTCCACCGGCGGCATGGACGAGCTGTACAAGTAA

>Unc-76::GFP (Unc-76-tagged fluorescent proteins fill cell bodies and axons and are excluded from the nucleus more than unlabelled GFP, as shown in Imai et al. 2009.)

ATGGCGGATCTGCGAGTACCGGACATTCCGCTCGCCTCGTGTGATGATGATGATATCGATAGTAATAAGAATTTGAGCAACCATTCATCAGACGAGAAACATCACTGCAACAGCAACAGCGACGAGGAACGTCTTCATGACGAGTTCTCTGGATCCCTTGAGGACCTTGTCGGCAACTTTGACGAAAAAATTGCGGCATGCCTGAAGGACCACGAGGTGACGACAGCGGATATTGCACCTGTGCAGATACGTACTCAAGAGGAAGTTATGAATGAAAGCCAAACATGGTGGACATTAACCGGAAACTTTGGAAACATTCAACCTCTCGACTTTGGAACCTCTTCGATATGTAAAAAGATGGCCGCAGCTCTGGACAGTGATTCATTGAAAGACGACGCATCTACACGCCGAAGTATGACAAATTCCGATGATGAGGATCTTTTACGACAACAAATGGATGTTCATCAAATGATTGGACATCATCATGGATCTACGGATACTGGTGGTGAAACACCTCCACAGACTGCTGATCAAGTTATCGAAGAAATTGATGAAATGTTACAGGTACCGGTCGCCACCATGGTGAGCAAGGGCGAGGAGCTGTTCACCGGGGTGGTGCCCATCCTGGTCGAGCTGGACGGCGACGTAAACGGCCACAAGTTCAGCGTGTCCGGCGAGGGCGAGGGCGATGCCACCTACGGCAAGCTGACCCTGAAGTTCATCTGCACCACCGGCAAGCTGCCCGTGCCCTGGCCCACCCTCGTGACCACCCTGACCTACGGCGTGCAGTGCTTCAGCCGCTACCCCGACCACATGAAGCAGCACGACTTCTTCAAGTCCGCCATGCCCGAAGGCTACGTCCAGGAGCGCACCATCTTCTTCAAGGACGACGGCAACTACAAGACCCGCGCCGAGGTGAAGTTCGAGGGCGACACCCTGGTGAACCGCATCGAGCTGAAGGGCATCGACTTCAAGGAGGACGGCAACATCCTGGGGCACAAGCTGGAGTACAACTACAACAGCCACAACGTCTATATCATGGCCGACAAGCAGAAGAACGGCATCAAGGTGAACTTCAAGATCCGCCACAACATCGAGGACGGCAGCGTGCAGCTCGCCGACCACTACCAGCAGAACACCCCCATCGGCGACGGCCCCGTGCTGCTGCCCGACAACCACTACCTGAGCACCCAGTCCGCCCTGAGCAAAGACCCCAACGAGAAGCGCGATCACATGGTCCTGCTGGAGTTCGTGACCGCCGCCGGGATCACTCTCGGCATGGACGAGCTGTACAAGTAA

>Unc-76::YFP(Venus)

ATGGCGGATCTGCGAGTACCGGACATTCCGCTCGCCTCGTGTGATGATGATGATATCGATAGTAATAAGAATTTGAGCAACCATTCATCAGACGAGAAACATCACTGCAACAGCAACAGCGACGAGGAACGTCTTCATGACGAGTTCTCTGGATCCCTTGAGGACCTTGTCGGCAACTTTGACGAAAAAATTGCGGCATGCCTGAAGGACCACGAGGTGACGACAGCGGATATTGCACCTGTGCAGATACGTACTCAAGAGGAAGTTATGAATGAAAGCCAAACATGGTGGACATTAACCGGAAACTTTGGAAACATTCAACCTCTCGACTTTGGAACCTCTTCGATATGTAAAAAGATGGCCGCAGCTCTGGACAGTGATTCATTGAAAGACGACGCATCTACACGCCGAAGTATGACAAATTCCGATGATGAGGATCTTTTACGACAACAAATGGATGTTCATCAAATGATTGGACATCATCATGGATCTACGGATACTGGTGGTGAAACACCTCCACAGACTGCTGATCAAGTTATCGAAGAAATTGATGAAATGTTACAGGTACCGGTCGCCACCATGGTGAGCAAGGGCGAGGAGCTGTTCACCGGGGTGGTGCCCATCCTGGTCGAGCTGGACGGCGACGTAAACGGCCACAAGTTCAGCGTGTCCGGCGAGGGCGAGGGCGATGCCACCTACGGCAAGCTGACCCTGAAGCTGATCTGCACCACCGGCAAGCTGCCCGTGCCCTGGCCCACCCTCGTGACCACCCTGGGCTACGGCCTGCAGTGCTTCGCCCGCCACCCCGACCACATGAAGCAGCACGACTTCTTCAAGTCCGCCATGCCCGAAGGCTACGTCCAGGAGCGCACCATCTTCTTCAAGGACGACGGCAACTACAAGACCCGCGCCGAGGTGAAGTTCGAGGGCGACACCCTGGTGAACCGCATCGAGCTGAAGGGCATCGACTTCAAGGAGGACGGCAACATCCTGGGGCACAAGCTGGAGTACAACTACAACAGCCACAACGTCTATATCACCGCCGACAAGCAGAAGAACGGCATCAAGGCCAACTTCAAGATCCGCCACAACATCGAGGACGGCGGCGTGCAGCTCGCCGACCACTACCAGCAGAACACCCCCATCGGCGACGGCCCCGTGCTGCTGCCCGACAACCACTACCTGAGCTACCAGTCCGCCCTGAGCAAAGACCCCAACGAGAAGCGCGATCACATGGTCCTGCTGGAGTTCGTGACCGCCGCCGGGATCACTCTCGGCATGGACGAGCTGTACAAGTA

>Human CD4::mCherry

ATGAACCGGGGAGTCCCTTTTAGGCACTTGCTTCTGGTGCTGCAACTGGCGCTCCTCCCAGCAGCCACTCAGGGAAAGAAAGTGGTGCTGGGCAAAAAAGGGGATACAGTGGAACTGACCTGTACAGCTTCCCAGAAGAAGAGCATACAATTCCACTGGAAAAACTCCAACCAGATAAAGATTCTGGGAAATCAGGGCTCCTTCTTAACTAAAGGTCCATCCAAGCTGAATGATCGCGCTGACTCAAGAAGAAGCCTTTGGGACCAAGGAAACTTCCCCCTGATCATCAAGAATCTTAAGATAGAAGACTCAGATACTTACATCTGTGAAGTGGAGGACCAGAAGGAGGAGGTGCAATTGCTAGTGTTCGGATTGACTGCCAACTCTGACACCCACCTGCTTCAGGGGCAGAGCCTGACCCTGACCTTGGAGAGCCCCCCTGGTAGTAGCCCCTCAGTGCAATGTAGGAGTCCAAGGGGTAAAAACATACAGGGGGGGAAGACCCTCTCCGTGTCTCAGCTGGAGCTCCAGGATAGTGGCACCTGGACATGCACTGTCTTGCAGAACCAGAAGAAGGTGGAGTTCAAAATAGACATCGTGGTGCTAGCTTTCCAGAAGGCCTCCAGCATAGTCTATAAGAAAGAGGGGGAACAGGTGGAGTTCTCCTTCCCACTCGCCTTTACAGTTGAAAAGCTGACGGGCAGTGGCGAGCTGTGGTGGCAGGCGGAGAGGGCTTCCTCCTCCAAGTCTTGGATCACCTTTGACCTGAAGAACAAGGAAGTGTCTGTAAAACGGGTTACCCAGGACCCTAAGCTCCAGATGGGCAAGAAGCTCCCGCTCCACCTCACCCTGCCCCAGGCCTTGCCTCAGTATGCTGGCTCTGGAAACCTCACCCTGGCCCTTGAAGCGAAAACAGGAAAGTTGCATCAGGAAGTGAACCTGGTGGTGATGAGAGCCACTCAGCTCCAGAAAAATTTGACCTGTGAGGTGTGGGGACCCACCTCCCCTAAGCTGATGCTGAGCTTGAAACTGGAGAACAAGGAGGCAAAGGTCTCGAAGCGGGAGAAGGCGGTGTGGGTGCTGAACCCTGAGGCGGGGATGTGGCAGTGTCTGCTGAGTGACTCGGGACAGGTCCTGCTGGAATCCAACATCAAGGTTCTGCCCACATGGTCCACCCCGGTGCAGCCAATGGCCCTGATTGTGCTGGGGGGCGTCGCCGGCCTCCTGCTTTTCATTGGGCTAGGCATCTTCTTCTGTGTCAGGACTAGTGTGAGCAAGGGCGAGGAGGATAACATGGCCATCATCAAGGAGTTCATGCGCTTCAAGGTGCACATGGAGGGCTCCGTGAACGGCCACGAGTTCGAGATCGAGGGCGAGGGCGAGGGCCGCCCCTACGAGGGCACCCAGACCGCCAAGCTGAAGGTGACCAAGGGTGGCCCCCTGCCCTTCGCCTGGGACATCCTGTCCCCTCAGTTCATGTACGGCTCCAAGGCCTACGTGAAGCACCCCGCCGACATCCCCGACTACTTGAAGCTGTCCTTCCCCGAGGGCTTCAAGTGGGAGCGCGTGATGAACTTCGAGGACGGCGGCGTGGTGACCGTGACCCAGGACTCCTCCCTGCAGGACGGCGAGTTCATCTACAAGGTGAAGCTGCGCGGCACCAACTTCCCCTCCGACGGCCCCGTAATGCAGAAGAAGACCATGGGCTGGGAGGCCTCCTCCGAGCGGATGTACCCCGAGGACGGCGCCCTGAAGGGCGAGATCAAGCAGAGGCTGAAGCTGAAGGACGGCGGCCACTACGACGCTGAGGTCAAGACCACCTACAAGGCCAAGAAGCCCGTGCAGCTGCCCGGCGCCTACAACGTCAACATCAAGTTGGACATCACCTCCCACAACGAGGACTACACCATCGTGGAACAGTACGAACGCGCCGAGGGCCGCCACTCCACCGGCGGCATGGACGAGCTGTACAAGTAA

For *Tyrp.a>2XGFP* sequence, refer to Racioppi et al. 2014.

**CRISPR sequences**

**G** + Target(N19)

Pax3/7.2.1: **G**TAGTGGAGATGGCAGCTCA

Pax3/7.4.1: **G**GACTAATAGAACTGACCGA

Control: **G**CTTTGCTACGATCTACATT

Published originally in Stolfi et al. 2014, this targets a sequence that does not exist in the *Ciona robusta* genome.

DenhT2: **G**CGAAATTGCTCGACGCGCT

This targets a ddN-specific *cis*-regulatory sequence upstream of *Dmbx,* but serves as a negative control for CRISPR in the neural plate borders, where *Dmbx* is not expressed.

Primers to validate Pax3/7 sgRNA efficacy by peakshift method:

Forward PCR primer: TGAAATGGCACACATTGCTG

Reverse PCR primer: TATCGGTTCAAATGGGTCCAC (also used to sequence Pax3/7.4.1 target site)

Expected band size: ~1500 bp

Internal primer to sequence Pax3/7.2.1 target site: GTGATGTATAACAGTTACAGTGC

>U6 promoter (Nishiyama and Fujiwara 2008)

TGGCGGGTGTATTAAACCACTAAACAAACAATTGCCCCAAGCTCTCTTCACAATTATAAACACTATAATGTTTGGACAAGAGATTAGCGTGGCTGTGACGAGAACTCTCAAAGGCTTGGTGTAATTGATATTTTATAAGAAGCAGATTAAACTTCAATACAGTTTACACCTCATTTACAAAAAATTGGCTGCCAAAATCGCTAATTTACACATATTTAAAACAATTTCAACACATATACACAGTATAGTATAACTGCATAATAAACAAATACATCTAACAGACACTCACTAATCTGCCATAACAAGCTTCAAAAACTTAAACTCGAAATTTTAGTGAATCTTTTTTTTTAAATGAATATTTTATTTAAAAAGTTAAAAATATTACAGTTCAGGTATAGGTTTACACCTAATCTTTAATAATCCGAACTAAATTTTAACTATTTAGAAACTTTTTCAACCAAAGTTTAAAAAAATAGATTTTTCGCACGCTAAAACTATCATTTACACAAAAAAATGCAACAAAATGCAGAAAAAAATTACATTAGAGTTTAGGTTAGTTACCTGCTAATCAATATAAACTAACTTCCCGCATAATATTCATCTAAAATTAGCAATAATCACGTTTTACGCTAAAATTTGTGTAAAACTAAACTTCGTCCTTTGTCAAGGAGAAAATTTGACTCAAAAGCTGCGCGCGCAGGGGAGATCCCCAAGCGAGTGTTTGTTACATCATAATCATGTGGAAAAATCCCCTAATAAGTAAAAATACATATTTTTTAATTTTGGGGGCAAATAAACCGCTTTTTATGTCTAAAAACGCCAAAAATGGATCGCGCGAGCCCAAAAACGCACAAATAACGTACAGACAGTGTCTCTGCGTACACAGACGGTATTTCCCCTTTAAATTGAGAACTAGACTTAAGCACGCTTATAAGTCTGGAAGGCATCCGATGGTATAGAT

>sgRNA F+E scaffold (Stolfi et al. 2014, based on Chen et al. 2013)

GTTTAAGAGCTATGCTGGAAACAGCATAGCAAGTTTAAATAAGGCTAGTCCGTTATCAACTTGAAAAAGTGGCACCGAGTCGGTGCTTTTTTT

>nls::Cas9[humanized]::nls (i.e. Cas9, Stolfi et al. 2014)

ATGGCTAGCCCCAAAAAGAAGAGGAAAGTGGACAAGAAGTATTCTATCGGACTGGACATCGGGACTAATAGCGTCGGGTGGGCCGTGATCACTGACGAGTACAAGGTGCCCTCTAAGAAGTTCAAGGTGCTCGGGAACACCGACCGGCATTCCATCAAGAAAAATCTGATCGGAGCTCTCCTCTTTGATTCAGGGGAGACCGCTGAAGCAACCCGCCTCAAGCGGACTGCTAGACGGCGGTACACCAGGAGGAAGAACCGGATTTGTTACCTTCAAGAGATATTCTCCAACGAAATGGCAAAGGTCGACGACAGCTTCTTCCATAGGCTGGAAGAATCATTCCTCGTGGAAGAGGATAAGAAGCATGAACGGCATCCCATCTTCGGTAATATCGTCGACGAGGTGGCCTATCACGAGAAATACCCAACCATCTACCATCTTCGCAAAAAGCTGGTGGACTCAACCGACAAGGCAGACCTCCGGCTTATCTACCTGGCCCTGGCCCACATGATCAAGTTCAGAGGCCACTTCCTGATCGAGGGCGACCTCAATCCTGACAATAGCGATGTGGATAAACTGTTCATCCAGCTGGTGCAGACTTACAACCAGCTCTTTGAAGAGAACCCCATCAATGCAAGCGGAGTCGATGCCAAGGCCATTCTGTCAGCCCGGCTGTCAAAGAGCCGCAGACTTGAGAATCTTATCGCTCAGCTGCCGGGTGAAAAGAAAAATGGACTGTTCGGGAACCTGATTGCTCTTTCACTTGGGCTGACTCCCAATTTCAAGTCTAATTTCGACCTGGCAGAGGATGCCAAGCTGCAACTGTCCAAGGACACCTATGATGACGATCTCGACAACCTCCTGGCCCAGATCGGTGACCAATACGCCGACCTTTTCCTTGCTGCTAAGAATCTTTCTGACGCCATCCTGCTGTCTGACATTCTCCGCGTGAACACTGAAATCACCAAGGCCCCTCTTTCAGCTTCAATGATTAAGCGGTATGATGAGCACCACCAGGACCTGACCCTGCTTAAGGCACTCGTCCGGCAGCAGCTTCCGGAGAAGTACAAGGAAATCTTCTTTGACCAGTCAAAGAATGGATACGCCGGCTACATCGACGGAGGTGCCTCCCAAGAGGAATTTTATAAGTTTATCAAACCTATCCTTGAGAAGATGGACGGCACCGAAGAGCTCCTCGTGAAACTGAATCGGGAGGATCTGCTGCGGAAGCAGCGCACTTTCGACAATGGGAGCATTCCCCACCAGATCCATCTTGGGGAGCTTCACGCCATCCTTCGGCGCCAAGAGGACTTCTACCCCTTTCTTAAGGACAACAGGGAGAAGATTGAGAAAATTCTCACTTTCCGCATCCCCTACTACGTGGGACCCCTCGCCAGAGGAAATAGCCGGTTTGCTTGGATGACCAGAAAGTCAGAAGAAACTATCACTCCCTGGAACTTCGAAGAGGTGGTGGACAAGGGAGCCAGCGCTCAGTCATTCATCGAACGGATGACTAACTTCGATAAGAACCTCCCCAATGAGAAGGTCCTGCCGAAACATTCCCTGCTCTACGAGTACTTTACCGTGTACAACGAGCTGACCAAGGTGAAATATGTCACCGAAGGGATGAGGAAGCCCGCATTCCTGTCAGGCGAACAAAAGAAGGCAATTGTGGACCTTCTGTTCAAGACCAATAGAAAGGTGACCGTGAAGCAGCTGAAGGAGGACTATTTCAAGAAAATTGAATGCTTCGACTCTGTGGAGATTAGCGGGGTCGAAGATCGGTTCAACGCAAGCCTGGGTACCTACCATGATCTGCTTAAGATCATCAAGGACAAGGATTTTCTGGACAATGAGGAGAACGAGGACATCCTTGAGGACATTGTCCTGACTCTCACTCTGTTCGAGGACCGGGAAATGATCGAGGAGAGGCTTAAGACCTACGCCCATCTGTTCGACGATAAAGTGATGAAGCAACTTAAACGGAGAAGATATACCGGATGGGGACGCCTTAGCCGCAAACTCATCAACGGAATCCGGGACAAACAGAGCGGAAAGACCATTCTTGATTTCCTTAAGAGCGACGGATTCGCTAATCGCAACTTCATGCAACTTATCCATGATGATTCCCTGACCTTTAAGGAGGACATCCAGAAGGCCCAAGTGTCTGGACAAGGTGACTCACTGCACGAGCATATCGCAAATCTGGCTGGTTCACCCGCTATTAAGAAGGGTATTCTCCAGACCGTGAAAGTCGTGGACGAGCTGGTCAAGGTGATGGGTCGCCATAAACCAGAGAACATTGTCATCGAGATGGCCAGGGAAAACCAGACTACCCAGAAGGGACAGAAGAACAGCAGGGAGCGGATGAAAAGAATTGAGGAAGGGATTAAGGAGCTCGGGTCACAGATCCTTAAAGAGCACCCGGTGGAAAACACCCAGCTTCAGAATGAGAAGCTCTATCTGTACTACCTTCAAAATGGACGCGATATGTATGTGGACCAAGAGCTTGATATCAACAGGCTCTCAGACTACGACGTGGACCACATCGTCCCTCAGAGCTTCCTCAAAGACGACTCAATTGACAATAAGGTGCTGACTCGCTCAGACAAGAACCGGGGAAAGTCAGATAACGTGCCCTCAGAGGAAGTCGTGAAAAAGATGAAGAACTATTGGCGCCAGCTTCTGAACGCAAAGCTGATCACTCAGCGGAAGTTCGACAATCTCACTAAGGCTGAGAGGGGCGGACTGAGCGAACTGGACAAAGCAGGATTCATTAAACGGCAACTTGTGGAGACTCGGCAGATTACTAAACATGTCGCCCAAATCCTTGACTCACGCATGAATACCAAGTACGACGAAAACGACAAACTTATCCGCGAGGTGAAGGTGATTACCCTGAAGTCCAAGCTGGTCAGCGATTTCAGAAAGGACTTTCAATTCTACAAAGTGCGGGAGATCAATAACTATCATCATGCTCATGACGCATATCTGAATGCCGTGGTGGGAACCGCCCTGATCAAGAAGTACCCAAAGCTGGAAAGCGAGTTCGTGTACGGAGACTACAAGGTCTACGACGTGCGCAAGATGATTGCCAAATCTGAGCAGGAGATCGGAAAGGCCACCGCAAAGTACTTCTTCTACAGCAACATCATGAATTTCTTCAAGACCGAAATCACCCTTGCAAACGGTGAGATCCGGAAGAGGCCGCTCATCGAGACTAATGGGGAGACTGGCGAAATCGTGTGGGACAAGGGCAGAGATTTCGCTACCGTGCGCAAAGTGCTTTCTATGCCTCAAGTGAACATCGTGAAGAAAACCGAGGTGCAAACCGGAGGCTTTTCTAAGGAATCAATCCTCCCCAAGCGCAACTCCGACAAGCTCATTGCAAGGAAGAAGGATTGGGACCCTAAGAAGTACGGCGGATTCGATTCACCAACTGTGGCTTATTCTGTCCTGGTCGTGGCTAAGGTGGAAAAAGGAAAGTCTAAGAAGCTCAAGAGCGTGAAGGAACTGCTGGGTATCACCATTATGGAGCGCAGCTCCTTCGAGAAGAACCCAATTGACTTTCTCGAAGCCAAAGGTTACAAGGAAGTCAAGAAGGACCTTATCATCAAGCTCCCAAAGTATAGCCTGTTCGAACTGGAGAATGGGCGGAAGCGGATGCTCGCCTCCGCTGGCGAACTTCAGAAGGGTAATGAGCTGGCTCTCCCCTCCAAGTACGTGAATTTCCTCTACCTTGCAAGCCATTACGAGAAGCTGAAGGGGAGCCCCGAGGACAACGAGCAAAAGCAACTGTTTGTGGAGCAGCATAAGCATTATCTGGACGAGATCATTGAGCAGATTTCCGAGTTTTCTAAACGCGTCATTCTCGCTGATGCCAACCTCGATAAAGTCCTTAGCGCATACAATAAGCACAGAGACAAACCAATTCGGGAGCAGGCTGAGAATATCATCCACCTGTTCACCCTCACCAATCTTGGTGCCCCTGCCGCATTCAAGTACTTCGACACCACCATCGACCGGAAACGCTATACCTCCACCAAAGAAGTGCTGGACGCCACCCTCATCCACCAGAGCATCACCGGACTTTACGAAACTCGGATTGACCTCTCACAGCTCGGAGGGGATGAGGGAGCTCCCAAGAAAAAGCGCAAGGTAGGTTAATGA

**CRISPR electroporation recipes (all per 700 ul total volume)**

*Pax3/7* CRISPR for peakshift

- 25 ul OSO-PCR, unpurified

- 25 ug Eef1a>Cas9

*Pax3/7* CRISPR in neural plate borders for IF (with CD4::mCherry)

- 35 ug FOG>Cas9

- 40 ug FOG>CD4::mCherry

- 25 ug U6>Pax3/7.2.1

- 25 ug U6>Pax3/7.4.1

Negative control in neural plate borders for IF (with CD4::mCherry)

- 35 ug FOG>Cas9

- 40 ug FOG>CD4::mCherry

- 50 ug 2mU6>Control

*Pax3/7* CRISPR in neural plate borders for IF scoring

- 25 ug FOG>Cas9

- 15 ug FOG>H2B::mCherry

- 40 ug U6>Pax3/7.2.1

- 40 ug U6>Pax3/7.4.1

Negative control in neural plate borders for IF scoring

- 25 ug FOG>Cas9

- 15 ug FOG>H2B::mCherry

- 80 ug 2mU6>Control

*Pax3/7* CRISPR in neural plate borders for neural tube closure assay

- 25 ug FOG>Cas9

- 15 ug FOG>H2B::mCherry

- 35 ug U6>Pax3/7.2.1

- 35 ug U6>Pax3/7.4.1

Negative control in neural plate borders for neural tube closure assay

- 25 ug FOG>Cas9

- 15 ug FOG>H2B::mCherry

- 70 ug 2mU6>DenhT2

*Pax3/7* CRISPR in neural plate borders for Tyrp.a>2XGFP scoring

- 25 ug FOG>Cas9

- 15 ug FOG>H2B::mCherry

- 70 ug Tyrp.a>2XGFP

- 40 ug U6>Pax3/7.2.1

- 40 ug U6>Pax3/7.4.1

Negative control in neural plate borders for Tyrp.a>2XGFP scoring

- 25 ug FOG>Cas9

- 15 ug FOG>H2B::mCherry

- 70 ug Tyrp.a>2XGFP

- 80 ug 2mU6>Control

*Pax3/7* CRISPR in A9.30 lineage

- 70 ug Fgf8/17/18>Cas9

- 25 ug Fgf8/17/18>H2B::mCherry

- 60 ug Dmbx -3490/-2159 + bpFOG>Unc-76::YFP

- 40 ug U6>Pax3/7.2.1

- 40 ug U6>Pax3/7.4.1

Negative control in A9.30 lineage

- 70 ug Fgf8/17/18>Cas9

- 25 ug Fgf8/17/18>H2B::mCherry

- 60 ug Dmbx -3490/-2159 + bpFOG>Unc-76::YFP

- 80 ug 2mU6>Control
