# Supplementary material for: Pax3/7 regulates neural tube closure and patterning in a non-vertebrate chordate
Source: Front Cell Dev Biol. 2022 Sep 12;10:999511. doi: 10.3389/fcell.2022.999511 (PMC9511217; doi:10.3389/fcell.2022.999511)
Supplement: Supplementary file 5 [file Table3.DOCX]

**
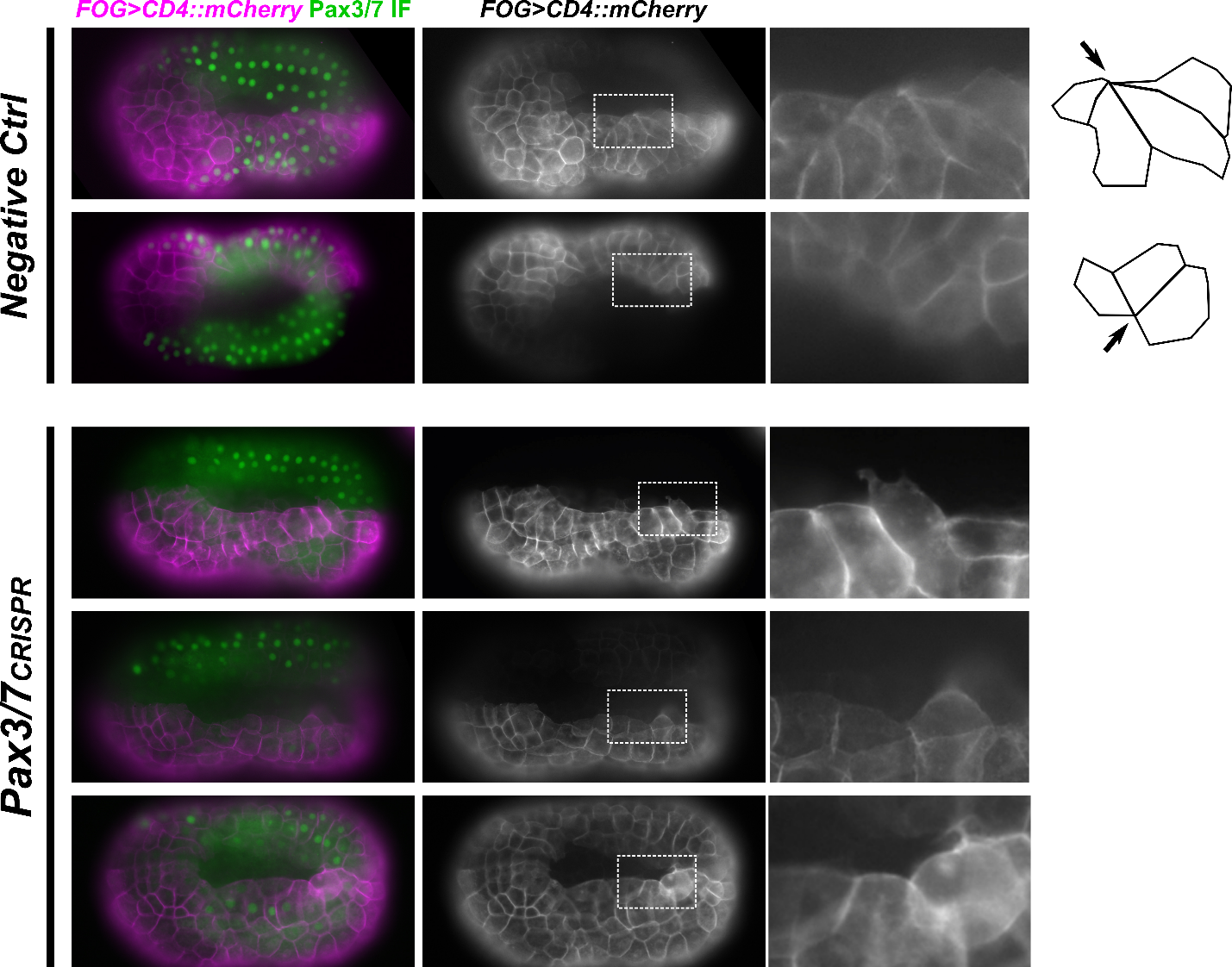
**

**Supplementary Figure 3. Lack of apical junction contractions and “zippering” in neural folds of Pax3/7 CRISPR embryos.**

Tissue-specific CRISPR/Cas9-mediated knockout of *Pax3/7,* performed in a/b-line cells using the *FOG>Cas9* plasmid and assayed by DP312 immunofluorescence at stage 16. *FOG>CD4::mCherry* counterstains cell membranes. To the right, magnified view and diagram showing the apical constrictions ahead of the “zipper” in negative control embryos, as described in Hashimoto and Munro 2019 and Hashimoto et al. 2015. Magnified view of *Pax3/7* CRISPR embryos shows lack of any such “zippering”. Embryos in top row of either condition are the same as in Figure 3c.
